# Assessment of Maternal and Neonatal SARS-CoV-2 Viral Load, Transplacental Antibody Transfer, and Placental Pathology in Pregnancies During the COVID-19 Pandemic

**DOI:** 10.1001/jamanetworkopen.2020.30455

**Published:** 2020-12-22

**Authors:** Andrea G. Edlow, Jonathan Z. Li, Ai-ris Y. Collier, Caroline Atyeo, Kaitlyn E. James, Adeline A. Boatin, Kathryn J. Gray, Evan A. Bordt, Lydia L. Shook, Lael M. Yonker, Alessio Fasano, Khady Diouf, Natalie Croul, Samantha Devane, Laura J. Yockey, Rosiane Lima, Jessica Shui, Juan D. Matute, Paul H. Lerou, Babatunde O. Akinwunmi, Aaron Schmidt, Jared Feldman, Blake M. Hauser, Timothy M. Caradonna, Denis De la Flor, Paolo D’Avino, James Regan, Heather Corry, Kendyll Coxen, Jesse Fajnzylber, David Pepin, Michael S. Seaman, Dan H. Barouch, Bruce D. Walker, Xu G. Yu, Anjali J. Kaimal, Drucilla J. Roberts, Galit Alter

**Affiliations:** 1 Department of Obstetrics, Gynecology and Reproductive Biology, Massachusetts General Hospital, Harvard Medical School, Boston; 2 Vincent Center for Reproductive Biology, Massachusetts General Hospital, Boston; 3 Department of Medicine, Brigham and Women’s Hospital, Harvard Medical School, Boston, Massachusetts; 4 Department of Obstetrics, Gynecology and Reproductive Biology, Beth Israel Deaconess Medical Center, Harvard Medical School, Boston, Massachusetts; 5 Center for Virology and Vaccine Research, Beth Israel Deaconess Medical Center, Harvard Medical School, Boston, Massachusetts; 6 Ragon Institute of MGH, MIT and Harvard, Harvard Medical School, Cambridge, Massachusetts; 7 Department of Obstetrics, Gynecology and Reproductive Biology, Brigham and Women’s Hospital, Harvard Medical School, Boston, Massachusetts; 8 Department of Pediatrics, Lurie Center for Autism, Massachusetts General Hospital, Harvard Medical School, Boston; 9 Department of Pediatrics, Massachusetts General Hospital, Harvard Medical School, Boston; 10 Department of Medicine, Massachusetts General Hospital, Harvard Medical School, Boston; 11 Department of Microbiology, Harvard Medical School, Boston, Massachusetts; 12 Pediatric Surgical Research Laboratories, Department of Surgery, Massachusetts General Hospital, Harvard Medical School, Boston; 13 Department of Pathology, Massachusetts General Hospital, Harvard Medical School, Boston

## Abstract

**Importance:**

Biological data are lacking with respect to risk of vertical transmission and mechanisms of fetoplacental protection in maternal severe acute respiratory syndrome coronavirus 2 (SARS-CoV-2) infection.

**Objective:**

To quantify SARS-CoV-2 viral load in maternal and neonatal biofluids, transplacental passage of anti–SARS-CoV-2 antibody, and incidence of fetoplacental infection.

**Design, Setting, and Participants:**

This cohort study was conducted among pregnant women presenting for care at 3 tertiary care centers in Boston, Massachusetts. Women with reverse transcription–polymerase chain reaction (RT-PCR) results positive for SARS-CoV-2 were recruited from April 2 to June 13, 2020, and follow-up occurred through July 10, 2020. Contemporaneous participants without SARS-CoV-2 infection were enrolled as a convenience sample from pregnant women with RT-PCR results negative for SARS-CoV-2.

**Exposures:**

SARS-CoV-2 infection in pregnancy, defined by nasopharyngeal swab RT-PCR.

**Main Outcomes and Measures:**

The main outcomes were SARS-CoV-2 viral load in maternal plasma or respiratory fluids and umbilical cord plasma, quantification of anti–SARS-CoV-2 antibodies in maternal and cord plasma, and presence of SARS-CoV-2 RNA in the placenta.

**Results:**

Among 127 pregnant women enrolled, 64 with RT-PCR results positive for SARS-CoV-2 (mean [SD] age, 31.6 [5.6] years) and 63 with RT-PCR results negative for SARS-CoV-2 (mean [SD] age, 33.9 [5.4] years) provided samples for analysis. Of women with SARS-CoV-2 infection, 23 (36%) were asymptomatic, 22 (34%) had mild disease, 7 (11%) had moderate disease, 10 (16%) had severe disease, and 2 (3%) had critical disease. In viral load analyses among 107 women, there was no detectable viremia in maternal or cord blood and no evidence of vertical transmission. Among 77 neonates tested in whom SARS-CoV-2 antibodies were quantified in cord blood, 1 had detectable immunoglobuilin M to nucleocapsid. Among 88 placentas tested, SARS-CoV-2 RNA was not detected in any. In antibody analyses among 37 women with SARS-CoV-2 infection, anti–receptor binding domain immunoglobin G was detected in 24 women (65%) and anti-nucleocapsid was detected in 26 women (70%). Mother-to-neonate transfer of anti–SARS-CoV-2 antibodies was significantly lower than transfer of anti-influenza hemagglutinin A antibodies (mean [SD] cord-to-maternal ratio: anti–receptor binding domain immunoglobin G, 0.72 [0.57]; anti-nucleocapsid, 0.74 [0.44]; anti-influenza, 1.44 [0.80]; *P* < .001). Nonoverlapping placental expression of SARS-CoV-2 receptors angiotensin-converting enzyme 2 and transmembrane serine protease 2 was noted.

**Conclusions and Relevance:**

In this cohort study, there was no evidence of placental infection or definitive vertical transmission of SARS-CoV-2. Transplacental transfer of anti-SARS-CoV-2 antibodies was inefficient. Lack of viremia and reduced coexpression and colocalization of placental angiotensin-converting enzyme 2 and transmembrane serine protease 2 may serve as protective mechanisms against vertical transmission.

## Key Points

**Question:**

What key biological characteristics of maternal severe acute respiratory syndrome coronavirus 2 (SARS-CoV-2) infection and placental function and pathology have implications for vertical transmission and neonatal protection?

**Findings:**

In this prospective cohort study including 127 pregnancies, there was no maternal viremia, placental infection, or vertical transmission of SARS-CoV-2. Compromised transplacental transfer of anti–SARS-CoV-2 antibodies with robust transfer of influenza-specific immunity and nonoverlapping placental expression of SARS-CoV-2 receptors angiotensin-converting enzyme 2 and transmembrane serine protease 2 were noted.

**Meaning:**

These findings suggest that, although low rates of maternal viremia and patterns of placental SARS-CoV-2 receptor distribution may underlie the rarity of vertical transmission, reduced transplacental transfer of anti–SARS-CoV-2 antibodies may leave neonates at risk for infection.

## Teaser

This cohort study examines maternal and neonatal severe acute respiratory syndrome coronavirus 2 viral load, transplacental antibody transfer, and placental pathology among pregnant women.

## Introduction

Recent data from the Centers for Disease Control and Prevention (CDC)^1^ suggest that pregnant women with severe acute respiratory syndrome coronavirus 2 (SARS-CoV-2) infection are more likely to require intensive care unit stay or mechanical ventilation than nonpregnant women of reproductive age. New data suggest that there is an increased risk of fetal^2^ and maternal^3^ death in the setting of maternal SARS-CoV-2 infection. While pregnancy-specific immunological and physiological changes may predispose women to increased morbidity in the setting of respiratory viruses,^4^ data are lacking regarding biological correlates of maternal disease severity in SARS-CoV-2 and have largely been extrapolated from nonpregnant populations or from pregnant women with SARS-CoV-1 or Middle East respiratory syndrome infection.^5,6,7^ Data regarding the maternal immune response, vertical transmission, and placental infection have been limited largely to case reports, small case series, and systematic reviews.^8,9,10,11,12,13,14,15,16,17,18,19,20^ Here, we report key biological data from a large prospective cohort study of SARS-CoV-2 infection in pregnancy regarding viral load, antibody response, transplacental antibody transfer, and placental pathology.

## Methods

This study was approved by institutional review boards at all participating centers. All participants provided written informed consent. This study is reported following the Strengthening the Reporting of Observational Studies in Epidemiology (STROBE) reporting guideline.

### Study Design

Pregnant women at 3 tertiary care centers in Boston, Massachusetts (Massachusetts General, Brigham and Women’s Hospital, and Beth Israel Deaconess Medical Center), were approached for enrollment in a coronavirus disease 2019 (COVID-19) pregnancy biorepository study starting April 2, 2020. Pregnant women were eligible for inclusion if they were aged 18 years or older, able to provide informed consent or had a health care proxy to do so, and diagnosed with, or at risk for, SARS-CoV-2 infection. Owing to wide community spread in Massachusetts during the study,^21^ all pregnant women presenting for hospital care were deemed at risk. Maternal confirmed SARS-CoV-2 infection was defined as nasopharyngeal swab reverse transcription–polymerase chain reaction (RT-PCR) test results positive for SARS-CoV-2. Neonates born to women with confirmed SARS-CoV-2 infection were tested by nasopharyngeal swab at age 24 hours.

### Participants and Procedures

Identification of eligible participants with SARS-CoV-2 infection and enrollment of controls is detailed in the eAppendix in the Supplement. Universal screening for SARS-CoV-2 among all pregnant women on admission to labor and delivery units (initiated on April 16, 2020 at Massachusetts General Hospital and Brigham and Women’s Hospital and on April 27, 2020 at Beth Israel Deaconess Medical Center) permitted the enrollment of a robust control population. The National Institutes of Health and Society for Maternal-Fetal Medicine criteria were used to define COVID-19 disease severity (eAppendix in the Supplement).^21,22^ Severe maternal morbidity was defined per CDC criteria,^23^ and severe neonatal morbidity was defined per Maternal-Fetal Medicine Units Network criteria^24,25^ (eAppendix in the Supplement). Nonpregnant women of reproductive age (18-45 years) hospitalized with confirmed SARS-CoV-2 infection and enrolled contemporaneously as part of a general adult cohort described previously^26^ were used as an additional comparison group.

### Sample Collection and Processing, SARS-CoV-2 Viral Load, and Antibody Quantification

Details on collection and processing of plasma, nasal and oropharyngeal swabs, saliva, sputum, and placenta, and comprehensive descriptions of methods for viral load quantification, enzyme-linked immunosorbent assay, and placental pathology are available in the eAppendix in the Supplement. SARS-CoV-2 viral load was quantified using the CDC 2019-nCoV_N1 primers and probe set.^27^ RNA was extracted from maternal and cord blood plasma and maternal respiratory specimens collected for research purposes; the clinical nasopharyngeal swab was not used for viral load quantification. Maternal blood and respiratory specimens collected in closest proximity to acute illness were used for viral load analyses. SARS-CoV-2 viral loads less than 40 RNA copies/mL were categorized as undetectable and set at 1.0 log_10_ RNA copies/mL.

Antibodies against SARS-CoV-2 receptor binding domain (RBD) on the S1 subunit of the spike protein and SARS-CoV-2 nucleocapsid (N) antigen were quantified using enzyme-linked immunosorbent assay. Quantification of antibody against the common influenza antigen hemagglutinin A (HA) was performed as a positive control. For transplacental antibody transfer analyses, paired maternal-cord blood samples from the delivery hospitalization were used.

### Placental Pathology

An experienced placental pathologist (D.J.R.) reviewed the slides and gross findings from all cases and controls, rendering diagnoses per the Amsterdam guidelines (eTable 1 in the Supplement).^28^ Placentas from participants with SARS-CoV-2 infection were tested for placental infection using RNA in situ hybridization, as previously described.^29,30^ A SARS-CoV-2 –positive lung section and known SARS-CoV-2–positive placental section^31^ were used as positive controls (eFigure 1 in the Supplement). A subset of 7 cases from patients who were mildly or severely ill were additionally examined for expression of the SARS-CoV-2 receptor angiotensin-converting enzyme 2 (ACE2) and the spike transmembrane serine protease 2 (TMPRSS2), which is required for viral cell entry,^32^ by immunohistochemistry.

### Outcomes

Primary outcomes in this study were quantification of SARS-CoV-2 viral load in maternal plasma, maternal respiratory fluids, and umbilical cord plasma; quantification of anti-SARS-CoV-2 immunoglobin (Ig) G and IgM antibodies in maternal and cord plasma; and presence of SARS-CoV-2 RNA in the placenta and evaluation of placental histopathology in cases and controls. The main secondary outcome was placental ACE2 and TMPRSS2 receptor expression.

### Statistical Analyses

Differences between participants with confirmed SARS-CoV-2 infection and those with RT-PCR results negative for SARS-CoV-2 with respect to demographic variables, viral load, antibody response, placental gene expression, and placental pathology were evaluated using appropriate tests (ie, parametric or nonparametric) with 2-sided *P* values. Continuous outcome measures were summarized as either mean (SD) or median (interquartile range [IQR]), as appropriate for the normality of the data. Associations between disease severity and factors of interest were analyzed in either dichotomized or ordinal fashion, using Pearson χ^2^ (or Fisher exact) test, or Spearman rank-based testing. Correlation analyses between maximum maternal viral load, antibody response, and COVID-19 severity were performed using Spearman rank-based testing. Differences between paired maternal and cord sera IgG and IgM were evaluated with Wilcoxon signed rank testing. Statistical significance was defined as *P* < .05; Bonferroni *P* value corrections were used for placental pathology analyses. Analyses were performed using Prism version 8 (GraphPad) and Stata/IC version 14.2 (StataCorp). The eAppendix in the Supplement contains additional description of statistical methods.

## Results

From April 2 through June 13, 2020, samples were obtained from 127 enrolled participants, 64 with RT-PCR results positive for SARS-CoV-2 and 63 with RT-PCR results negative for SARS-CoV-2. These included 88 completed mother-neonate dyads (47 with SARS-CoV-2 infection and 41 without SARS-CoV-2 infection), 4 women who delivered but did not have neonatal samples for analysis, 9 women who were still pregnant when the study ended, and 26 neonatal samples (placenta or umbilical cord blood) without matched maternal samples (eFigure 2 in the Supplement).

### Participant Characteristics

Maternal and neonatal demographic characteristics and outcomes for cases and controls are reported in the Table. Participant demographic characteristics for the nonpregnant cohort are reported in eTable 2 in the Supplement. Among 64 participants with SARS-CoV-2 infection, 23 (36%) were asymptomatic, 22 (34%) had mild disease, 7 (11%) had moderate disease, 10 (16%) had severe disease, and 2 (3%) had critical disease.^21,22^ A total of 9 patients (14%) were diagnosed in the second trimester, and 54 patients (86%) were diagnosed in the third trimester. There were 2 fetal or neonatal deaths in the SARS-CoV-2 positive group: one 35-week intrauterine fetal demise in an asymptomatic woman diagnosed with SARS-CoV-2 on presentation for management of intrauterine fetal demise, and one 22-week neonatal demise secondary to extreme prematurity in the setting of abruption and preterm labor in a symptomatic patient. No neonates born to women with confirmed SARS-CoV-2 infection had positive test results for SARS-CoV-2. Assay results for each participant are detailed in eTable 3 in the Supplement. Detailed information on severe maternal and neonatal morbidity is provided in eTable 4 and eTable 5 in the Supplement.

**Table.** Cohort Demographic Characteristics and Delivery Outcomes

Characteristic	SARS-CoV-2 status, No. (%)		*P* value
	Negative (n = 63)	Positive (n = 64)	
Maternal age, mean (SD), y	33.9 (5.4)	31.6 (5.6)	.02
Race			
Asian	6 (10)	0	<.001
Black	5 (8)	3 (5)	
White	44 (70)	26 (41)	
Other^a^	5 (8)	16 (25)	
>1 Race	0	7 (11)	
Unknown or not reported	3 (5)	12 (19)	
Ethnicity			
Hispanic or Latino	13 (20)	42 (66)	<.001
Not Hispanic or Latino	46 (73)	21 (33)	
Unknown or not reported	5 (8)	2 (3)	
Type of insurance			
Private	48 (76)	20 (32)	<.001
Public	15 (24)	43 (68)	
Unknown	0	1 (2)	
Pregravid BMI			
<18.5	1 (2)	0	.04
18.5-24.9	29 (46)	15 (23)	
25.0-29.9	15 (24)	23 (36)	
≥30.0	18 (29)	26 (41)	
Gestational weight gain, median (IQR), lb	28 (20-35)	21 (12-29)	.007
Gravidity, median (IQR), No.	2 (1-3)	3 (2-4)	.06
Parity, median (IQR), No.	1 (0-1)	1 (0-2)	.11
History of preterm birth	3 (5)	6 (9)	.36
Maternal comorbidities			
Chronic hypertension	1 (2)	3 (5)	.32
Diabetes or gestational diabetes	12 (19)	11 (17)	.79
BMI >30	18 (29)	26 (41)	.15
Asthma	8 (13)	7 (11)	.76
Other preexisting pulmonary condition	0	2 (3)	.16
Chronic kidney disease	0	0	NA
HIV	0	0	NA
IBD	0	0	NA
Thyroid disease	4 (6)	13 (21)	.02
Cancer	2 (3)	2 (3)	.99
Substance use within past year			
Alcohol	1 (1)	0	.53
Cigarettes or tobacco	5 (8)	0	.02
Marijuana	2 (3)	1 (2)	.55
Other vape	0	0	NA
Opioids	0	0	NA
Opioid replacement therapy	0	0	NA
Other	0	1 (2)	.32
Gestational age at delivery, median, (IQR), wk^b^	39.1 (38.3-39.7)	39 (37.4-40.1)	.14
Preterm delivery^c^	5 (8)	10 (18)	.11
Spontaneous	2 (40)	3 (30)	NA
Detectable SARS-CoV-2 antibodies (RBD or N)^d^			
IgG	1 (2.5)	26 (70)	<.001
IgM	1 (2.5)	16 (43)	
Labor^b^	38 (60)	47 (82)	.008
Mode of delivery^b^			.006
Vaginal	24 (38)	36 (63)	NA
Cesarean	39 (62)	21 (37)	
Preeclampsia/gestational hypertension^b^	13 (21)	15 (26)	.46
Infant sex^b^			
Male	34 (54)	26 (45)	.31
Female	29 (46)	31 (54)	
Birthweight, mean (SD), g^b^	3429.21 (597.34)	3072.28 (669.8)	.003
Fetal growth restriction	1 (2)	4 (6)	.06
Neonate SARS-CoV-2 screening result^e^			
Negative	NA	48(75)	NA
Not tested	NA	9 (14)	NA
Not delivered at time of analysis/pending test/unknown	NA	7 (11)	NA
Composite morbidity			
Maternal	2 (3)	9 (14)	.03
Neonatal^b^	5 (8)	14 (25)	.01
Neonatal death^f^	0	2 (4)	.16

Abbreviations: BMI, body mass index (calculated as weight in kilograms divided by height in meters squared); IBD, inflammatory bowel disease; Ig, immunoglobin; IQR, interquartile range; N, nucleocapsid; NA, not applicable; RBD, receptor binding domain; SARS-CoV-2, severe acute respiratory syndrome coronavirus 2.

^a^ Among participants with SARS-CoV-2, other race included Asian Indian (1 participant), Cape Verdean (2 participants), Dominican (4 participants), Dominican Hispanic (1 participant), Guatemalan (2 participants), Honduran (1 participant), not listed (1 participant), other (3 participants), and Salvadoran (3 participants). Among participants without SARS-CoV-2, other race included Salvadoran (2 participants) and other (3 participants).

^b^ At analysis, 57 participants with SARS-CoV-2 had delivered.

^c^ Preterm birth was defined as less than 37 weeks’ gestation and classified as spontaneous (eg, spontaneous labor, preterm premature rupture of the membranes) or indicated (eg, hypertensive disorders of pregnancy).

^d^ Forty mothers without SARS-CoV-2 and 37 mothers with SARS-CoV-2 had antibodies quantified for antibody transfer experiments. All participants with positive results for IgM to RBD also had positive IgG to RBD results. Of 12 participants with positive IgM to N results, 9 participants also had positive IgG to N results.

^e^ Forty-eight neonates (84% of neonates born to mothers with SARS-CoV-2 infection) were tested. Seven mothers with SARS-CoV-2 infection had not yet delivered. Thirteen neonates born to mothers diagnosed with SARS-CoV-2 earlier in pregnancy, but who tested negative for SARS-CoV-2 at the time of delivery were not clinically tested for SARS-CoV-2, per hospital infection control policies. Four of these neonates underwent a research assessment with quantification of viral load, which was negative.

^f^ Neonatal deaths detailed in severe neonatal morbidity eTable 5 in the Supplement.

### Maternal Disease Severity

Maternal disease severity was significantly associated with detectable respiratory viral load (5 of 45 women [11%] with mild disease; 6 of 19 women [32%] with severe disease; *P* = .04) (eTable 6 in the Supplement). Maternal disease severity was positively correlated with serum concentration of C-reactive protein (Spearman ρ = 0.56; *P* = .003) and alanine aminotransferase (ρ = 0.42; *P* = .004). Disease severity was negatively correlated with white blood cell count (ρ = −0.57; *P* < .001).

### Viral Load

**Figure 1.**
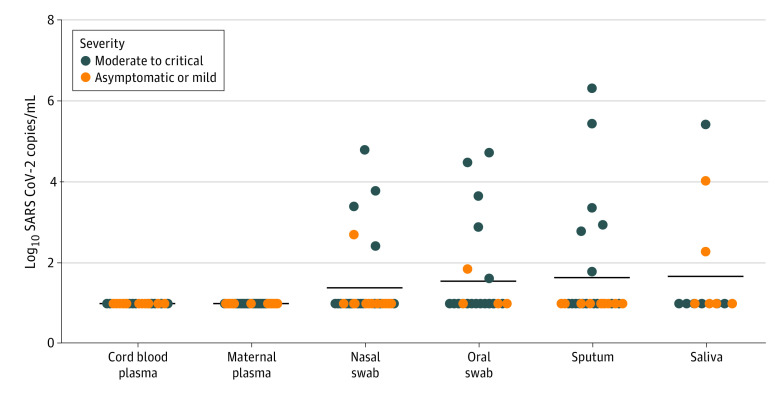
Severe Acute Respiratory Syndrome Coronavirus 2 (SARS-CoV-2) Viral Load in Maternal and Neonatal Biofluids and Tissues by Maternal Disease Severity The dot plot shows viral loads across samples by reverse transcription–polymerase chain reaction test results. Samples were analyzed in triplicate. No significant differences in viral load between any respiratory fluid were detected by Wilcoxon signed rank test.

SARS-CoV-2 viral load was quantified in 107 pregnant women (62 with SARS-CoV-2 infection and 45 without SARS-CoV-2 infection) and their neonates. Median (IQR) time from symptom onset to blood draw for viral load analysis was 13 (2-32) days. Median (IQR) time from symptom onset to collection of respiratory specimens for viral load analyses was 13 (1.75-31.75) days. There was no detectable viremia in any maternal or umbilical cord blood from 62 dyads with SARS-CoV-2 infection and 45 dyads without SARS-CoV-2 (Figure 1). Respiratory viral loads were quantified in 78 participants (44 with SARS-CoV-2 infection and 34 without SARS-CoV-2 infection). Among these, 11 women had detectable viral load in respiratory specimens (ie, nasal swab, oropharyngeal swab, saliva, or sputum). Among participants with RT-PCR confirmed SARS-CoV-2 infection, detectable viral load in maternal respiratory fluids was significantly associated with higher mean (SD) maternal anti-RBD IgG titers compared with 51 participants with undetectable viral load (0.79 [0.91] titers vs 0.29 [0.36] titers; *P* = .02) (eTable 7 in the Supplement). Among pregnant women with detectable viral load, sputum had the highest viral loads, followed by saliva, oral swab, and nasal swab. Viral load was not significantly correlated with any placental pathology among women with SARS-CoV-2 infection. Maternal viral load by time elapsed from SARS-CoV-2 diagnosis is depicted in eFigure 3 in the Supplement.

Compared with 11 nonpregnant women of reproductive age in the general adult cohort (age range, 22-42 years), there was no significant difference between mean (SD) respiratory viral load in pregnant and nonpregnant women (mean [SD], 3.8 [1.5] log_10_ copies/mL vs 4.8 [2.4] log_10_ copies/mL; *P* = .31). There was 1 case of viremia among women of reproductive age in the hospitalized nonpregnant cohort (plasma viral load of 2.4 log_10_ copies/mL detected 13 days after symptom onset).

### Transplacental Antibody Transfer

Antibody quantification was performed for 77 mother-neonate dyads, including 37 mothers with RT-PCR–confirmed SARS-CoV-2 infection and 40 mothers with RT-PCR results negative for SARS-CoV-2. Maternal and neonatal blood for transplacental antibody transfer analyses was drawn at the time of delivery admission. Median (IQR) time from symptom onset to blood draw for antibody quantification was 28.5 (9-44) days. Among mothers with SARS-CoV-2 infection, 24 (65%) had detectable anti-RBD IgG and 26 (70%) had detectable anti-N IgG (ρ = 0.71, *P* < .001). Among umbilical cords from mothers with SARS-CoV-2 infection, 23 (62%) had detectable anti-RBD IgG and 22 (59%) had detectable anti-N IgG.

**Figure 2.**
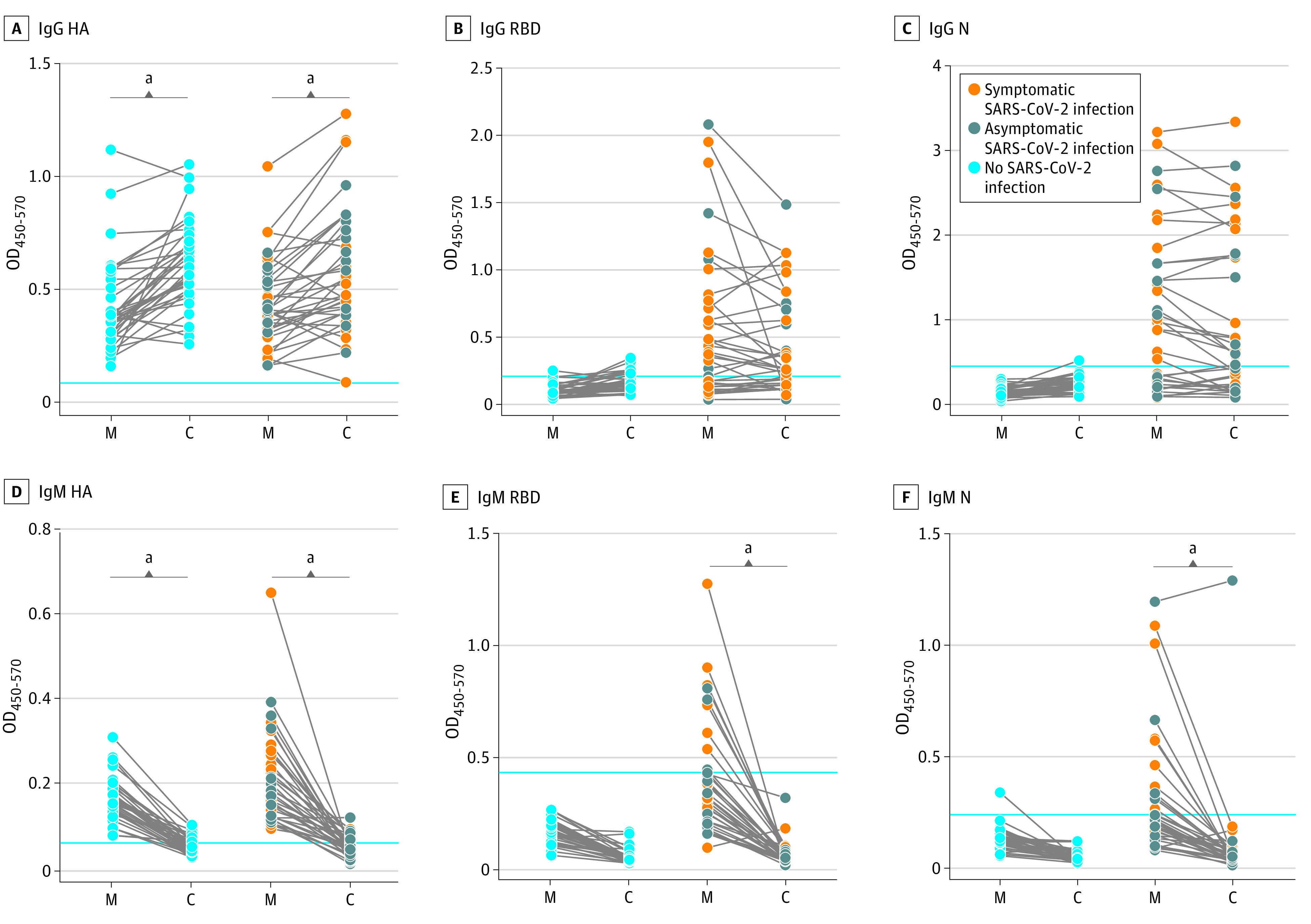
Maternal-Cord Transplacental Antibody Transfer of Anti-Influenza Hemagglutinin A (HA) and Anti–Severe Acute Respiratory Syndrome Coronavirus 2 (SARS-CoV-2) Antibodies The dot plots depict relative immunoglobuin (Ig) G or IgM titer against influenza HA, SARS-CoV-2 receptor binding domain (RBD), and SARS-CoV-2 nucleocapsid (N) present in maternal plasma (M) or matched umbilical cord blood (C). Data are represented as the optical density (OD) 450 value after background correction and are shown as the mean of 2 replicates. The blue lines represent the sum of the mean value of SARS-CoV-2 negative samples and 3 × the SD of those samples. Significance was determined by a Wilcoxon matched-pairs signed rank test. ^a^*P* < .001.

**Figure 3.**
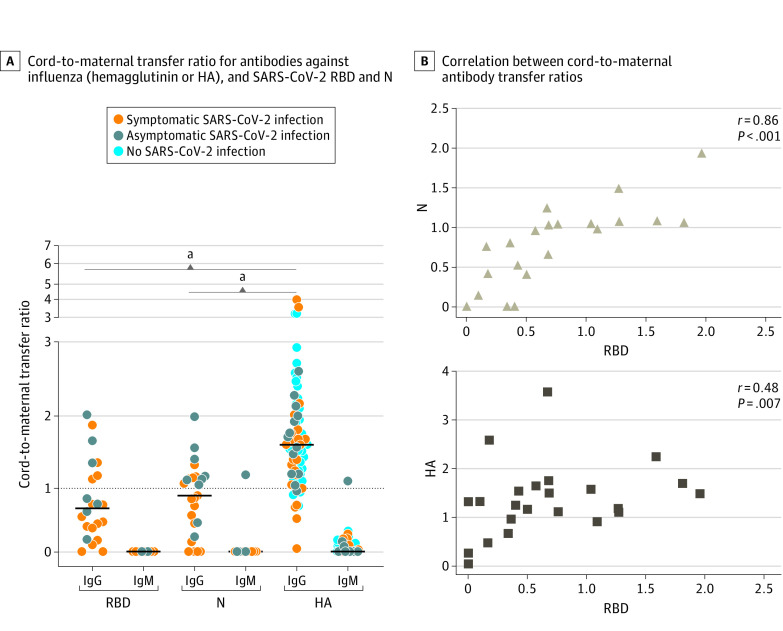
Cord-to-Maternal Transplacental Antibody Transfer Ratios for Influenza and Severe Acute Respiratory Syndrome Coronavirus 2 (SARS-CoV-2) A, Cord-to-maternal transfer ratio, calculated as (optical density [OD]_450-570_ cord)/(OD_450-570_ maternal) for antibodies against influenza hemagglutinin A (HA), and SARS-CoV-2 receptor binding domain (RBD) and nucleocapsid (N). Significance was assessed with a 1-way analysis of variance followed by Tukey post hoc testing to determine the source of significant differences in antibody transfer ratios among HA, RBD, and N. Ig indicates immunoglobin; black lines, means. B, Correlation between cord-to-maternal antibody transfer ratios for the indicated antibodies. Significance and ρ were determined by Spearman’s rank correlation. ^a^*P* < .001.

High transfer of influenza HA–specific antibody was observed regardless of maternal SARS-CoV-2 status (mean [SD] IgG HA transfer ratio: overall, 1.64 [1.37]; confirmed SARS-CoV-2, 1.44 [0.80]; *P* = .42). Compared with influenza, mean (SD) antibody transfer ratios were significantly reduced for anti–SARS-CoV-2 IgG against RBD (0.72 [0.57]; *P* < .001) and N (0.74 [0.44]; *P* < .001) (Figure 2A-C and Figure 3A). Maternal viral load was significantly negatively correlated with transplacental antibody transfer ratio of anti-RBD and anti-N IgG and negatively associated with cord blood anti-RBD and anti-N IgG titers (eTable 8 in the Supplement). Efficiency of antibody transfer (assessed by antibody transfer ratio) did not differ significantly between N and RBD, but mean (SD) antibody titers against N were significantly higher in the umbilical cord than those against RBD (0.77 [0.9] vs 0.24 [0.34]; *P* < .001). Cord blood titer of anti-RBD IgG was highly correlated with anti-N IgG (Spearman ρ = 0.86; *P* < .001) and with titers against HA (ρ = 0.48, *P* = .007, Figure 3B). Mean transfer ratios for IgG against RBD, N, and HA in preterm vs term neonates are depicted in eTable 9 in the Supplement.

As expected, IgM transfer across the placenta was rare (Figure 2D-F). N-specific IgM was observed in a single SARS-CoV-2 umbilical cord sample. The mother was asymptomatic, tests for IgM to RBD were negative in both maternal blood and cord plasma, and IgM to HA was detectable at equivalent levels in maternal and cord blood. The neonate had nasopharyngeal swab RT-PCR results negative for SARS-CoV-2, and was clinically well-appearing. This participant had high levels of anti-N IgM in maternal blood and placental pathology was notable for maternal vascular malperfusion lesions, plasma cell deciduitis, and villitis of unknown etiology, suggesting a compromised syncytiotrophoblast barrier, increasing potential placental leakiness. In addition, placental intervillous thrombi were noted, consistent with fetomaternal hemorrhage.

Maternal transplacental antibody transfer did not differ significantly by maternal disease severity or maternal medical comorbidities (ie, obesity, hypertension, or diabetes). Maternal and cord anti-SARS-CoV-2 antibody titers were significantly correlated with number of days from symptom onset (eFigure 4 and eFigure 5 in the Supplement). Compared with nonpregnant hospitalized women of reproductive age, there was no significant difference in mean (SD) antibody titers between pregnant and nonpregnant women (IgG RBD: 0.39 [0.52] vs 0.35 [0.40]; *P* = .63; IgG N: 0.88 [0.86] vs 0.51 [0.48]; *P* = .29). However, the mean (SD) time from symptom onset to antibody draw among nonpregnant women was shorter than in pregnant women (11 [5.1] days vs 28 [20.6] days).

### Placental Pathology

**Figure 4.**
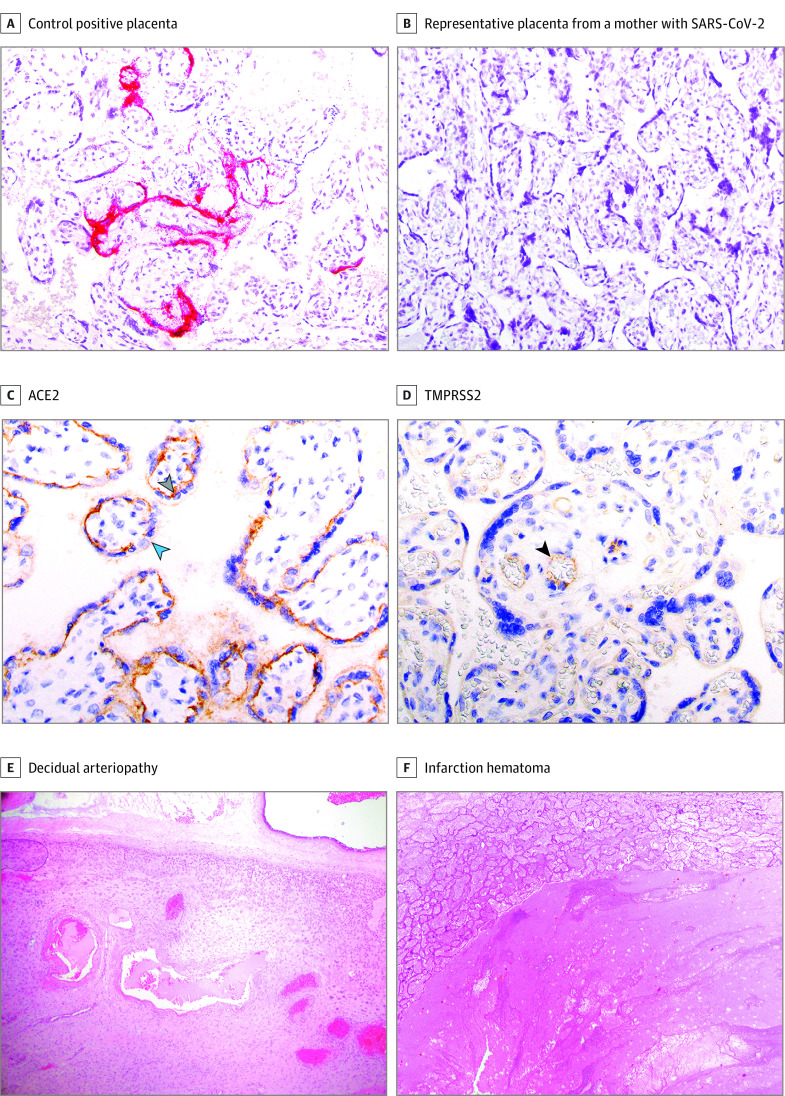
Placental Findings in Maternal Severe Acute Respiratory Syndrome Coronavirus 2 (SARS-CoV-2) Infection A and B: RNA in situ hybridization results at 20× original. Magenta red signal is visualized in the syncytiotrophoblast, with a complete lack of positivity in any stromal cells (including but not limited to Hofbauer cells) (A). C and D: SARS-CoV-2 receptor expression by immunohistochemistry at 40× original. C, Expression is restricted to the villous trophoblast with a polarity such that the highest expression is stromal side of the syncytiotrophoblast (gray arrow) with minimal to absent expression on the maternal vascular side (blue arrow). D, Weak expression limited to the villous endothelial cells (black arrow). Resident placental macrophages (Hofbauer cells) expressed neither angiotensin-converting enzyme 2 (ACE2) nor transmembrane serine protease 2 (TMPRSS2). E and F: Maternal vascular malperfusion (MVM) pathology. E, Hematoxylin and eosin stain at 4× original. F, Hematoxylin and eosin stain at 10× original.

Pathologic examinations were performed on 88 placentas, including 44 from women with SARS-CoV-2 and 44 from women without SARS-CoV-2. RNA in situ hybridization revealed no cases of SARS-CoV-2 RNA in the placenta. A known SARS-CoV-2–infected placenta was used as a positive control, and in situ hybridization did detect SARS-CoV-2 RNA in this placenta (Figure 4A and B). Expression of the SARS-CoV-2 entry receptors ACE2 and TMPRSS2 was examined via immunohistochemistry on placental tissue sections, and we identified membranous syncytiotrophoblastic ACE2 expression, with a strong bias of expression to the stromal side of the cell (Figure 4C). TMPRSS2 was weakly expressed primarily in the villous endothelium, not the syncytiotrophoblastic membrane (Figure 4D). There was no characteristic placental pathology in our SARS-CoV-2–exposed placentas (eTable 10 in the Supplement). However, maternal vascular malperfusion was noted in 16 of 44 (36%) SARS-CoV-2 exposed placentas and 8 of 44 (18%) unexposed placentas (*P* = .06) (Figure 4E and F). Among participants with confirmed SARS-CoV-2 infection, the odds of maternal vascular malperfusion lesions increased significantly with disease severity (odds ratio, 2.09 [95% CI, 1.11-3.97]; *P* = .02) (eTable 11 in the Supplement).

## Discussion

In this prospective cohort study of 127 pregnancies from a single city during the COVID-19 pandemic, we report zero cases of vertical transmission and no placental infection. We also report the absence of viremia in pregnant participants infected with SARS-CoV-2, relatively low frequency of detectable viral load in respiratory fluids, and a new finding of reduced efficiency of transplacental transfer of anti-SARS-CoV-2 antibodies. These data fill a substantial knowledge gap, point to the rarity of vertical transmission, and establish compromised SARS-CoV-2–specific immunity in the neonate. With only a handful of reported cases of vertical transmission,^10,13,17,18,19,20^ and the initial report from a prospectively-enrolled US registry demonstrating a vertical transmission rate of 1.1% (2 of 179 neonates),^33^ mechanisms of fetoplacental protection from SARS-CoV-2, and how these might impact neonatal immunity represent a critical area of investigation. Our study suggests low incidence of maternal viremia and nonoverlapping placental ACE2 and TMPRSS2 expression as potential mechanisms associated with protection against placental infection and vertical transmission in maternal COVID-19.

### Viral Load as a Potential Factor in Maternal Disease Severity and Neonatal Immunity

No women with confirmed SARS-CoV-2 infection had detectable viremia in our cohort. The reported incidence of SARS-CoV-2 viremia in the literature was 10% to 15% in 2 series of nonpregnant patients, including men and women.^34,35^ Rates of viremia as high as 27% were reported in a cohort of nonpregnant patients hospitalized during the same time period at the institutions included in this study, with the same median days from symptom onset to study blood draw and evaluated in the same laboratory, with the same assay.^26^ This highlights the sensitivity of the viral load assay, acuity of disease presentation at our hospitals during the study enrollment period, and ability of the assay to detect virus when present in plasma at the same median interval from symptom onset to blood draw. Thus, the absence of viremia in our cohort points to the potential for enhanced maternal viral control. The lack of maternal viremia is plausible given the low prevalence of placental infection reported in SARS-CoV-2 to date and points to low likelihood of viral placental seeding. Future investigations may provide insights into the mechanisms underlying the lower incidence of viremia observed here in the setting of pregnancy. Importantly, enhanced natural killer–cell and T-cell responses to influenza A virus have been noted during pregnancy,^36^ and are enriched within the placenta.^37^ These data suggest that cytotoxic cells may be poised to control viruses during pregnancy, providing a robust front line defense against pathogens.

Recent studies have reported reduced sensitivity of oropharyngeal swab,^38^ saliva,^39^ and anterior or lower nasal swab^40,41^ compared with nasopharyngeal swab for the detection of SARS-CoV-2. Among participants with symptomatic illness, the detection of viral load on research respiratory specimens was significantly more likely when specimens were collected closer to the clinical nasopharyngeal swab positive for SARS-CoV-2. Similar to studies in nonpregnant patients, viral load was associated with increased disease severity.^26,42^

### Transplacental Antibody Transfer and Positive Umbilical Cord IgM

Anti-RBD IgG and anti-N IgG transfer was significantly lower than for anti-HA IgG, and significantly lower than the expected cord-to-maternal antibody ratio of approximately 1.5 typically observed for pathogens including pertussis, influenza, and measles.^43,44,45^ Conversely, robust transfer of influenza-specific antibodies was noted, highlighting normal placental antibody transfer function. To our knowledge, this is the first report of reduced transplacental transfer of antibodies to SARS-CoV-2. These data point to a potential alteration in SARS-CoV-2–specific antibodies, which may result in compromised transfer. Whether altered transfer is related to infection-associated differences in SARS-CoV-2 antibody glycosylation or to differences in vaccine-elicited (HA) vs natural infection (SARS-CoV-2)–generated antibodies remains unclear. Recently reported findings that neonatal transferred antibodies may be short-lived^46^ raise further concern not only for poor initial transfer efficacy, but also for shorter durability of vertically transmitted immunity. Given data that demonstrate increased neonatal risk in the setting of reduced transplacental transfer secondary to native dengue virus infection,^47,48,49^ and maternal HIV (ie, reduced transfer of antibodies against tetanus, varicella-zoster virus, Epstein-Barr virus, measles, polio, and pertussis),^50,51,52^ it is certainly possible that reduced transplacental transfer of anti-SARS-CoV-2 antibodies increases risk of SARS-CoV-2 infection for neonates and infants. Recent data from the CDC indicate that infants aged 0 to 2 months comprise nearly 20% of all hospitalizations for SARS-CoV-2 infection among children aged 0 to 18 years.^53^

A single umbilical cord of a neonate born to a mother with confirmed SARS-CoV-2 infection had detectable IgM to SARS-CoV-2 N antigen. Given that placental pathology was suggestive of a damaged syncytiotrophoblast barrier and that we also observed high maternal levels of anti-N IgM and detectable anti-HA IgM in the cord at the same level as in maternal plasma, these findings likely represent aberrant transplacental transfer of IgM in the setting of placental compromise,^54^ rather than a specific elevation of IgM due to in utero infection. Although the finding of positive IgM has been interpreted as evidence of vertical transmission in prior reports,^10,11,46^ these findings raise the question of whether a more robust definition of vertical transmission is needed.

### Placental Pathology

No cases of suspected vertical transmission were observed, and no placental infections were diagnosed in our series of placentas exposed to SARS-CoV-2, suggesting the presence of intrinsic defenses against vertical transmission. The poorly overlapping expression of ACE2 and TMPRSS2 within the placenta may represent an unanticipated defense mechanism. Placental infection leading to fetal infection requires either infection of the villous syncytiotrophoblast or the villous stroma, followed by viral traversal of the villous endothelial cells to make contact with fetal blood. We report nonoverlapping expression of ACE2 and TMPRSS2 within the placenta, with polarized villous ACE2 expression restricted to the stromal side of the syncytiotrophoblast, and weak expression of TMPRSS2 in the villous endothelial cells. Hofbauer cells in the villous stroma express neither. This expression pattern suggests that the villi are somewhat protected from infection and may help explain why placental SARS-CoV-2 infection and vertical transmission is so rare. The nonoverlapping expression of ACE2 and TMPRSS2 and weaker expression of TMPRSS2 compared with ACE2 are consistent with recent single-cell RNA-Seq data reporting lack of coordinated cotranscription of ACE2 and TMPRSS2 in placentas predating the COVID-19 pandemic.^55^

### Limitations

This study has some limitations. The recruitment of controls as a convenience sample resulted in some demographic differences between cases and controls. Disproportionate COVID-19 disease burden in the Latinx community in the greater Boston area has been noted by our group,^56^ reflected in the demographic characteristics of our cases and controls. There was a higher rate of cesarean delivery among our controls, given that cesareans are often scheduled daytime procedures. It is both a strength and a limitation that our study necessarily examines transplacental antibody transfer in the setting of third trimester maternal infection with SARS-CoV-2, owing to the timing of the pandemic in Boston. The third trimester is typically regarded as the time when highest placental antibody transfer occurs,^57,58,59^ with most of these data from vaccinatable pathogens.^60,61,62,63^ While it is possible that antibody transfer may be lower in third trimester natural or native infection compared with second trimester infection, data are lacking in this regard. Comparing efficiency of antibody transfer in first, second, and third trimester native infection with SARS-CoV-2 will be an important area for future study, as women infected in the first and second trimester begin to deliver.

The timing of our study with the height of the first wave of the pandemic in Boston affords a unique opportunity to examine transfer of SARS-CoV-2 antibodies due to third-trimester native infection. The limited data available for Zika and Dengue virus infection in pregnancy demonstrate lower placental transfer ratios than have been described for influenza, pertussis, and measles vaccination^43,44,45^ but still higher ratios than we observed, ranging from 0.9 to approximately 1.2.^64,65,66,67^ Unlike our study, many studies on Zika and Dengue virus are unable to pinpoint the timing of maternal infection to a particular trimester, owing to their enrollment in endemic areas and use of antibody testing to determine infection. The large size of our cohort and the presence of robust contemporaneous controls with RT-PCR results negative for SARS-CoV-2 permit dissection of the impact of SARS-CoV-2 from other pandemic-related exposures that could influence maternal-fetal immune response^68,69,70^ is a strength of this study.

## Conclusions

This report of maternal viral load, transplacental antibody transmission, and placental pathology in 127 pregnancies during the SARS-CoV-2 pandemic provides needed data about maternal viral control, reduced transplacental transfer of anti–SARS-CoV-2 antibodies, and lack of vertical transmission in mother-neonate dyads. These findings can immediately inform clinical care and vaccine development and deployment strategies to maximize benefit for pregnant women and their neonates.

## Notes

Supplement. **eAppendix.** Supplementary Methods **eTable 1.** Placental Pathology Categories **eFigure 1.** SARS-CoV-2 RNA in Situ Hybridization **eFigure 2.** Participant Enrollment **eTable 2.** Participant Demographics for Nonpregnant Adult Cohort **eTable 3.** Experimental Assays Performed per Participant **eTable 4.** Severe Maternal Morbidity **eTable 5.** Severe Neonatal Morbidity **eTable 6.** Participant Characteristics, Viral Loads, Antibody Quantification, and Placental Pathology Stratified by Maternal Disease Severity **eFigure 3.** Maternal Viral Load by Time Elapsed From SARS-CoV-2 Diagnosis **eTable 7.** Detectable Viral Load and Maternal and Neonatal Antibody **eTable 8.** Correlations Between Maternal and Neonatal Antibody Titers, Transplacental Antibody Transfer, and Maximum Maternal Viral Load **eTable 9.** Antibody Transfer Ratio in Full-Term vs Preterm Gestations **eFigure 4.** Maternal Antibody Titers by Days From Symptom Onset **eFigure 5.** Cord Antibody Titers by Days from Maternal Symptom Onset **eTable 10.** Placental Pathology by Maternal SARS-CoV-2 Status **eTable 11.** Placental Pathology by Maternal SARS-CoV-2 Severity **eReferences**
