# Supplementary material for: Assessment of Maternal and Neonatal SARS-CoV-2 Viral Load, Transplacental Antibody Transfer, and Placental Pathology in Pregnancies During the COVID-19 Pandemic
Source: JAMA Netw Open. 2020 Dec 22;3(12):e2030455. doi: 10.1001/jamanetworkopen.2020.30455 (PMC7756241; doi:10.1001/jamanetworkopen.2020.30455)
Supplement: Supplement. — eAppendix. Supplementary Methods eTable 1. Placental Pathology Categories eFigure 1. SARS-CoV-2 RNA in Situ Hybridization eFigure 2. Participant Enrollment eTable 2. Participant Demographics for Nonpregnant Adult Cohort eTable 3. Experimental Assays Performed per Participant eTable 4. Severe Maternal Morbidity eTable 5. Severe Neonatal Morbidity eTable 6. Participant Characteristics, Viral Loads, Antibody Quantification, and Placental Pathology Stratified by Maternal Disease Severity eFigure 3. Maternal Viral Load by Time Elapsed From SARS-CoV-2 Diagnosis eTable 7. Detectable Viral Load and Maternal and Neonatal Antibody eTable 8. Correlations Between Maternal and Neonatal Antibody Titers, Transplacental Antibody Transfer, and Maximum Maternal Viral Load eTable 9. Antibody Transfer Ratio in Full-Term vs Preterm Gestations eFigure 4. Maternal Antibody Titers by Days From Symptom Onset eFigure 5. Cord Antibody Titers by Days from Maternal Symptom Onset eTable 10. Placental Pathology by Maternal SARS-CoV-2 Status eTable 11. Placental Pathology by Maternal SARS-CoV-2 Severity eReferences [file jamanetwopen-e2030455-s001.pdf]

Supplemental Online Content

Edlow AG, Li JZ, Collier AY, et al. Assessment of maternal and neonatal SARS-CoV-2 viral load, transplacental antibody transfer, and placental pathology in pregnancies during the COVID-19 pandemic. *JAMA Netw Open*. 2020;3(12):e2030455. doi:10.1001/jamanetworkopen.2020.30455

eAppendix. Supplementary Methods
eTable 1. Placental Pathology Categories
eFigure 1. SARS-CoV-2 RNA in Situ Hybridization
eFigure 2. Participant Enrollment
eTable 2. Participant Demographics for Nonpregnant Adult Cohort
eTable 3. Experimental Assays Performed per Participant
eTable 4. Severe Maternal Morbidity
eTable 5. Severe Neonatal Morbidity
eTable 6. Participant Characteristics, Viral Loads, Antibody Quantification, and Placental Pathology Stratified by Maternal Disease Severity
eFigure 3. Maternal Viral Load by Time Elapsed From SARS-CoV-2 Diagnosis
eTable 7. Detectable Viral Load and Maternal and Neonatal Antibody
eTable 8. Correlations Between Maternal and Neonatal Antibody Titers, Transplacental Antibody Transfer, and Maximum Maternal Viral Load
eTable 9. Antibody Transfer Ratio in Full-Term vs Preterm Gestations
eFigure 4. Maternal Antibody Titers by Days From Symptom Onset
eFigure 5. Cord Antibody Titers by Days from Maternal Symptom Onset
eTable 10. Placental Pathology by Maternal SARS-CoV-2 Status
eTable 11. Placental Pathology by Maternal SARS-CoV-2 Severity
eReferences

This supplemental material has been provided by the authors to give readers additional information about their work.

© 2020 Edlow AG et al. *JAMA Network Open.*

eAppendix. Supplementary Methods

Identification of Eligible Patients

Eligible SARS-CoV-2 positive patients were identified in one of three ways: (1) by dedicated study clinician (obstetrician, nurse practitioner or nurse midwife) present on the Labor and Delivery unit enrolling for the study; (2) via lists and automatic flags generated in the electronic medical record (EMR) of SARS-CoV-2 positive pregnant patients including all patients receiving inpatient or outpatient care at the participating institutions; (3) via consults to the antepartum service for pregnant patients with SARS-CoV-2 admitted to other services in the hospital (uniformly implemented for all positive pregnant patients not already cared for by the obstetrics team). Participants positive for SARS-CoV-2 were either admitted for evaluation and care for SARS-CoV-2-related illness, or were admitted for delivery and identified as SARS-CoV-2 positive on universal screening or by prior documented positive RTqPCR of nasopharyngeal swab RNA during pregnancy. Participants negative for SARS-CoV-2 participants were enrolled as a convenience sample, recruited from patients presenting on the same days as the enrolled positive cases, and likely to deliver during daytime hours. This was to facilitate study feasibility, as overnight delivery sample collection was prioritized for participants positive for SARS-CoV-2. Demographic and clinical outcomes data were abstracted from the electronic medical record for participants and their neonates, using REDCap electronic data capture tools.[1]

Sample Collection

© 2020 Edlow AG et al. *JAMA Network Open.*

Samples were collected at the time of acute illness if patient was hospitalized, and again at the time of delivery for already-enrolled patients positive for SARS-CoV-2. Acute samples were utilized if available for viral load analyses, delivery samples were utilized for all maternal-to-cord blood antibody transfer studies (thus there was no significant time discordance between maternal sample draw and delivery for the transfer analyses). For those patients who were not hospitalized for SARS-CoV-2-related illness and were instead found to have SARS-CoV-2 on admission to Labor and Delivery (symptomatic and asymptomatic), samples were collected as early as possible in relation to the diagnosis. Sample collection protocols have been described in a previous publication[2] and are summarized below.

Blood Plasma

Blood from pregnant and non-pregnant women was collected by venipuncture into EDTA tubes. Umbilical cord blood was collected immediately after delivery. The umbilical cord was wiped clean and blood was drawn directly from the vein using a syringe and transferred to EDTA vacutainer tubes. Blood was centrifuged at 1000g for 10 min at room temperature. Plasma was aliquoted into cryogenic vials and stored at -80°C.

Nasal and Oropharyngeal Swabs

Sterile nylon flocked swabs (Copan Diagnostics 518CS01) were rotated in each nostril or used to swab the oropharynx, and then placed in a 15 mL falcon tube prefilled with 3 mL of PBS. Swabs were stored at 4°C until processing, at which time they were mixed in PBS by pipetting, aliquoted into cryogenic vials and stored at -80°C.

Saliva and Sputum

Participants were directed to not eat or drink 30 minutes prior to saliva or sputum collection. Sputum was collected only in participants with productive cough. Saliva/sputum was collected in sterile specimen cups and stored at 4°C until processing. Saliva was diluted 1:1 with 10mM dithiothreitol (SigmaAldrich D0632) in cold PBS and mixed well by pipetting. Aliquots were stored in cryogenic vials at 80°C.

Placenta

For histopathological examination, placentas were fixed in formalin, weighed, examined grossly, and sectioned/stained as described below.

Viral Load Quantification by RTqPCR

Plasma, respiratory secretions and swab fluids were centrifuged at approximately 21,000 x g for 2 hours at 4°C. The supernatant was removed and TRIzol-LS™ Reagent (ThermoFisher) was added to the pellets and then incubated on ice, followed by chloroform (MilliporeSigma). The mixtures were separated by centrifugation at 21,000 x g for 15 minutes at 4°C, and subsequently the aqueous layer was removed and treated with an equal volume of isopropanol (Sigma). GlycoBlue™ Coprecipitant (ThermoFisher) and 100 µL 3M Sodium Acetate (Life Technologies) were added to each sample and incubated on dry ice until frozen. RNA was pelleted by centrifugation at 21,000 x g for 45 minutes at 4°C. The supernatant was discarded and the RNA was washed with cold 70% ethanol. The RNA was resuspended in DEPC-treated water (ThermoFisher). Each reaction contained extracted RNA, 1X TaqPath™ 1-Step RT-qPCR Master Mix, CG (ThermoFisher), the CDC N1 forward and reverse primers, and probe.[3] Viral copy numbers were quantified using N1 qPCR standards in 16-fold

© 2020 Edlow AG et al. *JAMA Network Open.*

dilutions to generate a standard curve. The assay was run in triplicate for each sample and two non-template control (NTC) wells were included as negative controls. Quantification of the Importin-8 (IPO8) housekeeping gene RNA level was performed to determine the quality of respiratory sample collection.[4-6] An internal virion control (RCAS) was spiked into each sample and quantified to determine the efficiency of RNA extraction and qPCR amplification.[7] SARS-CoV-2 viral loads below 40 RNA copies/mL were categorized as undetectable and set at 1.0 $\log_{10}$ RNA copies/mL.

Anti- SARS-CoV-2 Antibody and Anti-influenza (HA) antibody quantification in matched maternal and umbilical cord plasma by Enzyme-linked Immunosorbent Assay (ELISA)

Antibodies against SARS-CoV-2 receptor binding domain (RBD) on the S1 subunit of the Spike protein and SARS-CoV-2 Nucleocapsid (N) antigen were quantified using ELISA. Quantification of antibody against the common influenza antigen HA was performed to evaluate the relative efficiency of transplacental antibody transfer for RBD/N versus HA. ELISA plates were coated with 500 ng/mL per well of SARS-CoV-2 RBD, SARS-CoV-2 N (Aalto Bio Reagents), or a mix of three influenza HA (A/Michigan/45/2015, B/Phuket/3073/2013, A/Singapore/INFIMH-16-0019/2016; Immune Technology Corp.). Plates were incubated for 30 minutes at room temperature and washed in wash buffer (0.05% Tween-20, 400 mM NaCl, 50 mM Tris, pH 8.0). Plates were blocked with a 1% BSA solution then washed again with wash buffer. Serum samples were diluted 1:100 and added to the plates. Plates were incubated at 37°C for 30 minutes. After incubation, plates were washed, and anti-human IgG or anti-

© 2020 Edlow AG et al. *JAMA Network Open.*

human IgM coupled to horseradish peroxidase (HRP) (Bethyl Laboratories) was added for detection. Plates were incubated for 30 minutes at room temperature and washed. The ELISA was developed with TMB and stopped with sulfuric acid. The signal was read at 450 nm and background corrected from a reference wavelength of 570.

Placental Histopathology

Formalin-fixed placentas were examined by an experienced placental pathologist (DJR) and histopathologic diagnoses were rendered in categories (eTable 1), following the Amsterdam guidelines.[8] SARS-CoV-2 positive cases were tested for placental infection by SARS-CoV-2 using RNA in-situ hybridization (RNAish). One full-thickness section of placental parenchyma from each case was cut at 5 microns for RNAish. RNAish was performed using the RNAscope 2.5 HD Reagent Kit (RED) (ACD Bio, # 322350) and following the manufacturer's instructions for standard conditions as recommended for human lung and placenta. The slide processing method has been previously described.[9,10] Both lung and a known SARS-CoV-2 positive placenta were used as positive controls (eFigure 1). Placenta sections were then hybridized with a pre-designed probe spanning COVID-19 sense strand mRNA to detect the coronavirus (V-ncov2019-S, #848561) for 2h at 40°C, processed for standard signal amplification steps, and a red chromogen development was performed using the RNAscope 2.5 HD (Red) detection Kit (ACD Bio, #322360). The slides were then counterstained in 25% hematoxylin (Dako, #S2302) for 30s, air-dried overnight and coverslipped with EcoMount.

© 2020 Edlow AG et al. *JAMA Network Open.*

A subset of seven cases including mild (3) and severely-ill (4) participants were examined for expression of the SARS-CoV-2 receptor ACE2, the spike serine proteinase TMPRSS2 (required for viral cell entry), and the viral Nucleocapsid (N) protein by immunohistochemistry (IHC). Five-micron sections were prepared from full thickness placental parenchyma for IHC. IHC was performed using an automated stainer (Bond-III; Leica Microsystems Bannockburn, IL) with ACE2 Monoclonal Antibody (clone CL4035 [1:15,000], Thermo Fisher Scientific, Waltham, MA), TMPRSS2 antibody (Clone PA5-83286 [1:1,000] Thermo-Invitrogen, Carlsbad, CA) , and SARS Nucleocapsid Protein Antibody (clone NB100-56576 [1:300], Novus Biologicals, Littleton, CO) in accordance with the manufacturer's recommendations.

Definitions of severe maternal and neonatal morbidity

In accordance with the CDC and American College of Obstetricians and Gynecologists (ACOG) definitions of severe maternal morbidity, the composite severe maternal morbidity outcome was defined as any (or multiple) of the following: (1) Acute Respiratory Distress Syndrome (ARDS); (2) Acute myocardial infarction (MI); (3) Amniotic Fluid Embolism (AFE); (4) Congestive Heart Failure (CHF); (5) Sepsis/Shock; (6) Hemorrhage; (7) Eclampsia; (8) Disseminated Intravascular Coagulation (DIC); (9) Hysterectomy; (10) ICU admission; (11) Acute Renal Failure (ARF); (12) Assisted Ventilation (intubation); (13) Death. There were no maternal deaths. In accordance with Maternal-Fetal Medicine Units Network criteria,[11,12] the composite severe neonatal morbidity outcome was defined as any (or multiple) of the following: (1) Respiratory Distress Syndrome; (2) Transient Tachypnea of the Newborn; (3) Sepsis; (4) Assisted

© 2020 Edlow AG et al. *JAMA Network Open.*

Ventilation; (5) Seizure; (6) Grade 3 or 4 intraventricular hemorrhage; (7) Necrotizing enterocolitis; (8) NICU admission; (9) CPAP or supplemental O2.

Definitions of COVID-19 severity

COVID severity was defined according to NIH and Society for Maternal-Fetal Medicine criteria[13,14]:

- Symptomatic or pre-symptomatic disease or presumptive infection is defined as a positive COVID-19 test result with no symptoms.
- Mild disease is defined as flu-like symptoms, such as fever, cough, myalgias, and anosmia without dyspnea, shortness of breath, or abnormal chest imaging.
- Moderate disease is defined by evidence of lower respiratory tract disease with clinical assessment (dyspnea, pneumonia on imaging, abnormal blood gas results, refractory fever of 39.0 °C /102.2°F or greater not alleviated with acetaminophen) while maintaining an oxygen saturation of greater than 93% on room air at sea level.
- Severe disease is defined by a respiratory rate greater than 30 breaths per minute, hypoxia with oxygen saturation less than or equal to 93%, a ratio of arterial partial pressure of oxygen to fraction of inspired oxygen of less than 300, or greater than 50% lung involvement on imaging.
- Critical disease is defined as multi-organ failure or dysfunction, shock, or respiratory failure requiring mechanical ventilation or high-flow nasal cannula.

Of note, while the Royal College of Obstetricians and Gynecologists (RCOG) has designated pregnant women > 28 weeks as a particularly high-risk group due to the

© 2020 Edlow AG et al. *JAMA Network Open.*

observation that most pregnant women with COVID-19 were hospitalized in the third trimester of pregnancy or peripartum,[15,16] Society for Maternal-Fetal Medicine criteria do not consider gestational age > 28 weeks to be a criteria for severity *per se*.

Statistical Analyses

Differences between SARS-CoV-2 positive cases and controls with respect to demographic variables, viral load, antibody response, and placental pathology were evaluated using appropriate tests (parametric or non-parametric, based on normality of data distribution, with two-sided p-values. These tests included Pearson's chi-squared test, Fisher's exact test, Student's t-test, Mann-Whitney U test, Wilcoxon matched pairs signed rank testing, and Spearman rank-based correlations. To further elucidate associations between COVID-19 disease severity and factors of interest, SARS-CoV-2 positive patients were dichotomized into asymptomatic or mild cases (notated as "mild") versus moderate, severe, or critical cases (notated as "severe"), following criteria set forth by the NIH and SMFM,[13,14] or were analyzed in an ordinal fashion based on their disease severity. Within cases, associations between COVID-19 disease severity and viral load and antibody levels were examined in a continuous fashion with non-parametric testing (Mann Whitney U test); viral load was further categorized as detectable versus undetectable and compared with Fisher's exact or Chi square tests.

Correlation analyses between maximum maternal viral load, antibody response, and COVID-19 severity were performed using Spearman rank-based testing. For paired maternal and neonatal cord blood antibody analyses, data were analyzed using Wilcoxon matched pairs signed rank test. Differences in antibody transfer ratios

© 2020 Edlow AG et al. *JAMA Network Open.*

between RBD, N and flu HA were determined using one-way ANOVA with Tukey's post-hoc testing to determine the source of differences, $p<0.05$ Based on the effect size observed for reduced transplacental antibody transfer of RBD and N, we had > 95% statistical power to detect the observed significant differences with an alpha set at <0.05. The impact of maternal medical comorbidities on the relationship between COVID-19 severity and viral load, antibody transfer, or placental pathology was examined using stratified analyses. Statistical significance was defined as a p-value of <0.05; Bonferroni p-value corrections were utilized for placental pathology analyses (correcting for two comparisons, $p<0.025$). Analyses were performed using GraphPad Prism 8 and Stata/IC, version 14.2. Data were analyzed by AGE, KEJ, JZL, CA, EAB, GA, AYC and DJR.

Antibody quantification was performed in 77 dyads with matched maternal and neonatal cord blood (37 SARS-CoV-2 positive, 40 SARS CoV-2 negative). Placental pathology was performed on a subset of 88 placentas (44 SARS-CoV-2 positive, 44 negative). Not all participants for whom placenta was available had a completed set of matched maternal and umbilical cord blood, accounting for the difference between the number of placentas analyzed versus ELISAs performed. Viral load quantification was performed throughout the study period, ultimately including a larger subset of patients as matched samples were not required for these analyses (N=107).

© 2020 Edlow AG et al. *JAMA Network Open.*

eTable 1. Placental Pathology Categories

Category	Example of diagnoses	Grade
**Fetal vascular malperfusion (FVM)**	Vascular thrombi, stem villous obliteration, intramural fibrin deposition, villous stromal-vascular karyorrhexis, avascular villi	Per Khong et al [8]
**Maternal vascular malperfusion (MVM)**	Placental hypoplasia (weight $\leq$ 10th percentile for gestational age), accelerated villous maturation, distal villous hypoplasia, decidual arteriopathy, placental infarct, abruption, increased perivillous fibrin	Low grade - <3 findings<br>High grade - $\geq$ 3 findings
**Inflammatory (Infl)**	Villitis of unknown etiology (VUE), chronic deciduitis/plasma cell deciduitis, chronic chorioamnionitis, chronic histiocytic intervillositis (CHI), Hofbauer cell hyperplasia, eosinophilic T-cell chorionic vasculitis	Low grade – not high grade<br>High grade - VUE per Khong et al [8], CHI, any diffuse or marked pathology.
**Infectious (Infe)**	Acute chorioamnionitis, acute villitis, specific infectious chronic villitis	Low grade – not high grade<br>High grade - acute chorioamnionitis any maternal or fetal inflammation stage 2 or grade 2 per Khong et al [8], more than focal acute villitis, any infectious chronic villitis
**Findings associated with placental hypoxia (Hypo)**	Villous edema, meconium pigment, chorangiosis, fetal normoblastemia	Low grade – mild, focal, or patchy pathology<br>High grade – strong/robust, multifocal or diffuse pathology
**Gross abnormalities (Gross)**	Abnormal insertion of the umbilical cord or membranes, excessively long or short umbilical cords, abnormal number of umbilical cord coils, accessory placental lobes, abnormal shape of the disk	Not graded
**Other (Other)**	Massive perivillous fibrin deposition/maternal floor infarct (MPFD/MFI), chorangioma, chorangiomatosis, delayed villous maturation, intervillous/subchorionic/septal thrombus, adherent myometrial fibers	Low grade – single, mild, focal, or patchy pathology<br><br>High grade – MPFD/MFI, strong/robust, multifocal or diffuse pathology

© 2020 Edlow AG et al. *JAMA Network Open.*

eFigure 1. SARS-CoV-2 RNA in Situ Hybridization

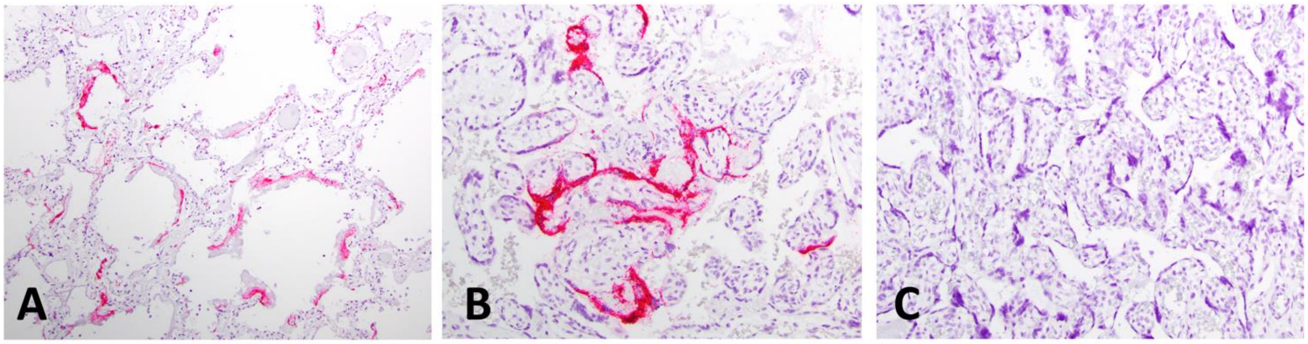

RNA in situ hybridization (RNAish) was performed using the RNAscope 2.5 HD Reagent Kit (RED) (ACD Bio, # 322350). Both a COVID-19 positive autopsy lung section (A) and a known SARS-CoV-2 positive placental section (B) were used as controls. Magneta red staining indicates SARS-CoV-2 RNA positivity. 0/44 placentas from SARS-CoV-2 positive women contained detectable SARS-CoV-2 RNA in a full-thickness section. (C) depicts a representative negative placenta from a SARS-CoV-2 positive participant.

© 2020 Edlow AG et al. *JAMA Network Open.*

eFigure 2. Participant Enrollment

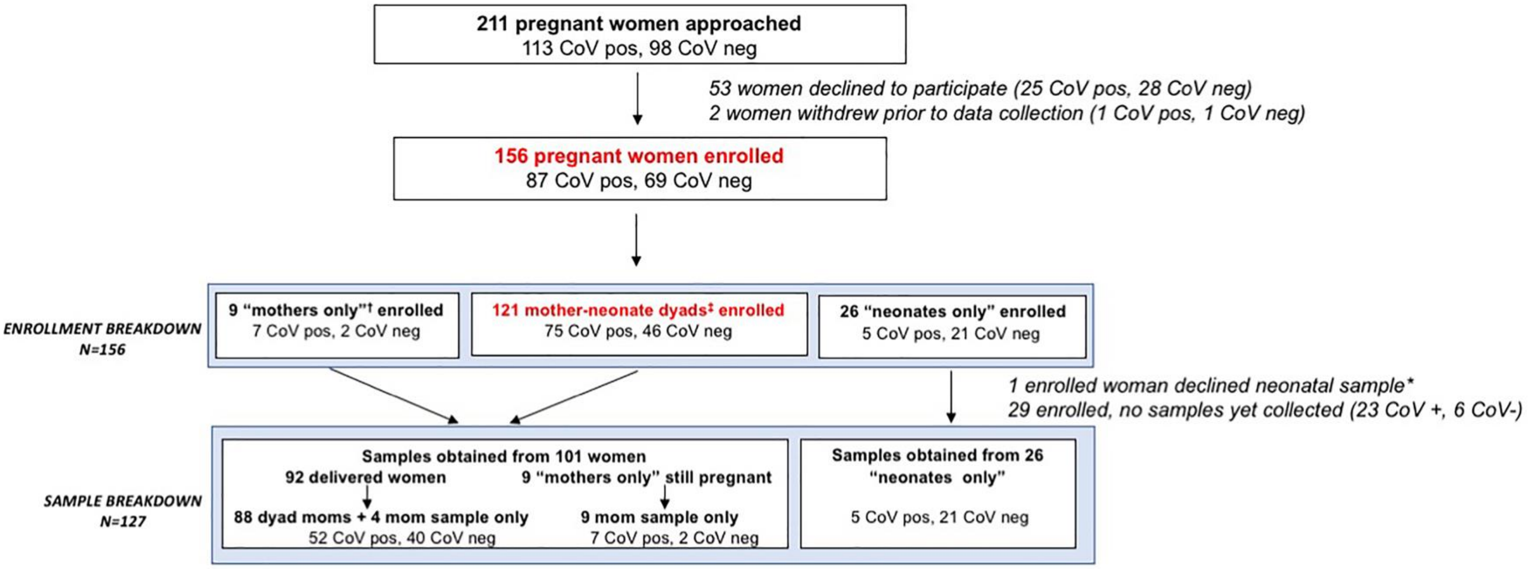

*Neonatal samples are defined as placenta, umbilical cord blood. Those designated "neonates only" had only placenta and/or cord blood collected but no maternal sample.
†"Mothers only" are those mothers still pregnant who already contributed a maternal sample (maternal blood and/or respiratory sample, including saliva/sputum, nasal swab, oropharyngeal swab)
‡"Dyad" is defined as a maternal-neonatal pair with at least one maternal (blood and/or respiratory) and one neonatal (placenta and/or cord blood) sample collected.
CoV pos = SARS-CoV-2 positive; CoV neg = SARS-CoV-2 negative.

© 2020 Edlow AG et al. *JAMA Network Open.*

eTable 2. Participant Demographics for Nonpregnant Adult Cohort

**Demographics and Participant Characteristics**	**Whole hospitalized cohort, SARS-CoV-2 positive (N=88)**	**Hospitalized women of reproductive age, SARS-CoV-2 positive (N=11)**
**Age, years (mean, SD)**	57.0 (16.0)	32.6 (6.7)
**Sex (Female, %)**	38%	100%
**Days from symptom onset to sample collection (median)**	13	12
**Race[a]**		
Asian	2 (2%)	0 (0%)
Black/African American	13 (15%)	0 (0%)
White	31 (35%)	6 (55%)
Other	26 (30%)	1 (9%)
More than one race	9 (10%)	2 (18%)
Unknown/not reported	7 (8%)	2 (18%)
**Ethnicity**		
Hispanic or Latino	33 (38%)	3 (27%)
Not Hispanic or Latino	48 (55%)	6 (55%)
Unknown/Not Reported	7 (8%)	2 (18%)
**BMI (pre-gravid)**		
Underweight (<18.5)	1 (1%)	0 (0%)
Normal (18.5-24.9)	16 (18%)	1 (9%)
Overweight (25.0-29.9)	32 (36%)	1 (9%)
Obese (>-30.0)	39 (44%)	9 (82%)
**Medical Comorbidities**		
Chronic HTN	47 (53%)	4 (36%)
Diabetes/GDM	36 (41%)	2 (18%)
BMI >30	39 (44%)	9 (82%)
Asthma	9 (10%)	4 (36%)
Other preexisting pulmonary condition	16 (18%)	3 (27%)
Chronic kidney disease	1 (1%)	0 (0%)
HIV	2 (2%)	0 (0%)
IBD	0 (0%)	0 (0%)
Thyroid disease	0 (0%)	0 (0%)
Cancer	2 (2%)	0 (0%)

© 2020 Edlow AG et al. *JAMA Network Open.*

eTable 3. Experimental Assays Performed per Participant

Participant number	COVID Status	COVID Severity[a]	Viral Load Analysis	Antibody Analysis	Placental Pathology[b]
1	COVID positive	severe	ND		HYPO, OTHER
2	COVID positive	critical	ND		
3	COVID positive	critical	**Nasal Swab**: ND (4/10), 2.4 (4/16), 2.1 (4/24); **Oral Swab**: 4.7 (4/10), ND (4/16), 2.0 (4/24); **Urine**: ND (4/10), ND (4/16), ND (4/24); **Maternal Plasma**: ND (4/10), ND (4/24); **Sputum:** 5.5 (4/16), ND (4/24)	**Maternal Plasma**: IgG RBD, IgG HA; **Cord Plasma**: IgG HA	MVM, INFLAM
4	COVID positive	moderate	ND	**Maternal Plasma**: IgG RBD, IgG N, IgM RBD, IgM N, IgG HA; **Cord Plasma**: IgG RBD, IgG N, IgG HA	INFEC, OTHER
5	COVID positive	asymptomatic/mild	**Nasal Swab:** ND; **Sputum:** 1.8; **Urine:** ND; **Maternal Plasma:** ND		FVM, INFLAM, OTHER
6	COVID positive	severe	ND	**Maternal Plasma**: IgG RBD, IgG N, IgG HA; **Cord Plasma**: IgG HA	MVM, HYPO, OTHER
7	COVID positive	asymptomatic/mild	**Nasal Swab:** 3.4; **OP Swab:** 4.5; **Urine:** ND; **Plasma:** ND; **Placenta:** ND	**Maternal Plasma:** ND; **Cord Plasma**: IgG HA	MVM, OTHER
8	COVID positive	moderate	ND		
9	COVID negative	N/A, negative	ND		
10	COVID negative	N/A, negative	ND		NONE
11	COVID negative	N/A, negative	ND		INFEC
12	COVID negative	N/A, negative	ND	**Maternal Plasma:** IgG HA; **Cord Plasma:** IgG HA	GROSS
13	COVID negative	N/A, negative	ND		GROSS
14	COVID negative	N/A, negative	ND	**Maternal Plasma**: ND; **Cord Plasma**: IgG HA	FVM, INFLAM
15	COVID negative	N/A, negative	ND	**Maternal Plasma**: ND; **Cord Plasma**: IgG HA	MVM, INFLAM

© 2020 Edlow AG et al. *JAMA Network Open.*

Participant number	COVID Status	COVID Severity	Viral Load Analysis	Antibody Analysis	Placental Pathology
16	COVID negative	N/A, negative	ND	**Maternal Plasm**a: IgG HA; **Cord Plasma**: IgG HA	MVM, GROSS, OTHER
17	COVID negative	N/A, negative	ND	**Maternal Plasma**: ND; **Cord Plasma**: IgG HA	MVM, GROSS, OTHER
18	COVID positive	asymptomatic/mild	ND	**Maternal Plasma**: IgG N, IgG HA; **Cord Plasma:** IgG HA	INFEC, INFLAM
19	COVID negative	N/A, negative	ND	**Maternal Plasma**: IgG HA; **Cord Plasma**: IgG HA	
20	COVID negative	N/A, negative	ND	**Maternal Plasma**: IgG HA; **Cord Plasma**: IgG HA	INFLAM, GROSS
21	COVID negative	N/A, negative	ND	**Maternal Plasma**: IgG HA; **Cord Plasma**: IgG HA	
22	COVID negative	N/A, negative	ND	**Maternal Plasma**: IgG HA; **Cord Plasma**: IgG HA	HYPO, OTHER
23	COVID positive	severe	ND		MVM, GROSS
24	COVID positive	severe	**Maternal Plasma:** ND; **CB Plasma:** ND; **Sputum:** 6.3; **Nasal Swab:** 3.8; **Oral Swab:** 2.9; **Placenta:** ND	**Maternal Plasma**: IgG HA; **Cord Plasma:** IgG HA	INFEC, HYPO, OTHER
25	COVID positive	asymptomatic/mild	ND		NONE
26	COVID negative	N/A, negative	ND	**Maternal Plasma**: IgG HA; **Cord Plasma**: IgG HA	NONE
27	COVID negative	N/A, negative	ND	**Maternal Plasma:** IgG HA; **Cord Plasma**: IgG HA	NONE
28	COVID positive	asymptomatic/mild	ND	**Maternal Plasma**: IgG RBD, IgG N, IgG HA, IgM HA; **Cord Plasma**: IgG RBD, IgG N, IgG HA	FVM, INFEC, INFLAM, HYPO
29	COVID positive	asymptomatic/mild	ND	**Maternal Plasma**: IgG RBD, N IgG, IgM N, IgG HA; **Cord Plasma**: IgG RBD, IgG N, IgG HA	MVM, INFLAM, OTHER
30	COVID negative	N/A, negative	ND	**Maternal Plasma**: ND; **Cord Plasma**: IgG HA	NONE

© 2020 Edlow AG et al. *JAMA Network Open.*

Participant number	COVID Status	COVID Severity	Viral Load Analysis	Antibody Analysis	Placental Pathology
31	COVID positive	asymptomatic/mild	ND	**Maternal Plasma**: IgG RBD, IgG N, IgM N, IgG HA, IgM HA; **Cord Plasma**: IgG RBD, IgG N, IgG HA	MVM, OTHER
32	COVID negative	N/A, negative	ND	**Maternal Plasma**: IgG HA; **Cord Plasma**: IgG HA	
33	COVID negative	N/A, negative	ND	**Maternal Plasma**: IgG HA, IgM HA; **Cord Plasma**: IgG HA	
34	COVID positive	moderate	ND	**Maternal Plasma**: IgG RBD, IgG N, IgM RBD, IgG HA, IgM HA; **Cord Plasma**: IgG RBD, IgG N, IgG HA	NONE
35	COVID negative	N/A, negative	ND	**Maternal Plasma:** IgG HA; **Cord Plasma**: IgG HA	FVM, INFLAM, OTHER
36	COVID positive	asymptomatic/mild	**Maternal Plasma:** ND; **CB Plasma:** ND; **Saliva:** 2.3; **Nasal Swab:** ND; **Oral Swab:** 1.9; **Placenta:** ND	**Maternal Plasma**: IgG RBD, IgG N, IgM RBD, IgM N, IgG HA; **Cord Plasma**: IgG RBD, IgG N, IgG HA	INFEC, HYPO, GROSS
37	COVID positive	asymptomatic/mild	**Maternal Plasma:** ND; **CB Plasma:** ND; **Saliva:** 4.0; **Nasal Swab:** 2.7; **Placenta:** ND	**Maternal Plasma**: IgG RBD, IgG N, IgG HA; **Cord Plasma**: IgG N, IgG HA	MVM, FVM, INFEC, HYPO
38	COVID negative	N/A, negative	ND	**Maternal Plasma:** IgG HA; **Cord Plasma:** IgG HA	
39	COVID positive	severe	**Mat Plasma:** ND; **CB Plasma:** ND; **Saliva:** 5.4; **Nasal Swab:** 4.8; **Oral Swab:** 1.6; **Placenta:** ND	**Maternal Plasma**: IgG HA; **Cord Plasma**: IgG HA	MVM, OTHER
40	COVID negative	N/A, negative	ND	**Maternal Plasma**: IgG HA; **Cord Plasma**: IgG HA	MVM, HYPO
41	COVID negative	N/A, negative	ND	**Maternal Plasma**: IgG HA; **Cord Plasma**: IgG HA	GROSS, OTHER
42	COVID negative	N/A, negative	ND	**Maternal Plasma**: IgG HA; **Cord Plasma**: IgG HA	INFEC, HYPO, GROSS, OTHER
43	COVID positive	asymptomatic/mild			MVM, GROSS, OTHER

© 2020 Edlow AG et al. *JAMA Network Open.*

Viral Load Analysis	Antibody Analysis	Placental Pathology	Viral Load Analysis	Antibody Analysis	Placental Pathology
44	COVID positive	asymptomatic/mild	ND	**Maternal Plasma**: IgG RBD, IgG N, IgM RBD, IgM N, IgG HA, IgM HA; **Cord Plasma**: IgG RBD, IgG N, IgG HA	MVM, INFEC, HYPO, OTHER
45	COVID negative	N/A, negative	ND	**Maternal Plasma**: IgM N, IgG HA, IgM HA; **Cord Plasma**: IgG HA	FVM, INFEC, INFLAM, HYPO
46	COVID negative	N/A, negative	ND	**Maternal Plasma**: IgG HA; **Cord Plasma**: IgG HA	INFLAM, HYPO, GROSS, OTHER
47	COVID negative	N/A, negative	ND	**Maternal Plasma**: IgG HA, IgM HA; **Cord Plasma**: IgG HA	
48	COVID negative	N/A, negative	ND	**Maternal Plasma**: IgG HA; **Cord Plasma**: IgG HA	
49	COVID positive	asymptomatic/mild	ND	**Maternal Plasma**: IgG RBD, IgG N; **Cord Plasma**: IgG RBD, IgG N, IgG HA	MVM, INFLAM, GROSS
50	COVID positive	asymptomatic/mild	ND		INFLAM, OTHER
51	COVID positive	asymptomatic/mild	ND	**Maternal Plasma:** IgG RBD; **Cord Plasma**: ND	MVM, INFLAM
52	COVID negative	N/A, negative	ND	**Maternal Plasma**: IgG HA; **Cord Plasma**: IgG HA	
53	COVID negative	N/A, negative	ND	**Maternal Plasma**: IgG HA; **Cord Plasma**: IgG HA	
54	COVID positive	COVID positive/severe	ND		
55	COVID negative	N/A, negative	ND		FVM, HYPO
56	COVID positive	asymptomatic/mild	ND		INFLAM
57	COVID negative	N/A, negative	ND	**Maternal Plasma:** IgG HA; **Cord Plasma**: IgG HA	
58	COVID negative	N/A, negative	ND	**Maternal Plasma:** IgG HA, IgM HA; **Cord Plasma:** IgG HA	

© 2020 Edlow AG et al. *JAMA Network Open.*

Viral Load Analysis	Antibody Analysis	Placental Pathology	Viral Load Analysis	Antibody Analysis	Placental Pathology
59	COVID negative	N/A, negative	ND	**Maternal Plasma:** IgG HA; **Cord Plasma**: IgG HA	
60	COVID negative	N/A, negative	ND	**Maternal Plasma**: IgG HA; **Cord Plasma**: ND	MVM, FVM, HYPO
61	COVID negative	N/A, negative	ND	**Maternal Plasma**: IgG HA; **Cord Plasma**: IgG HA	GROSS
62	COVID negative	N/A, negative	ND	**Maternal Plasma**: ND; **Cord Plasma**: IgG HA	MVM, FVM, HYPO
63	COVID negative	N/A, negative	ND	**Maternal Plasma**: IgG HA; **Cord Plasma**: IgG HA	
64	COVID negative	N/A, negative	ND	**Maternal Plasma**: IgG HA; **Cord Plasma:** IgG HA	NONE
65	COVID negative	N/A, negative	ND	**Maternal Plasma**: IgG HA; **Cord Plasma**: IgG HA (both neonates)	INFLAM, GROSS
66	COVID positive	asymptomatic/mild	ND	**Maternal Plasma**: IgG HA, IgM HA; **Cord Plasma**: IgG HA	FVM
67	COVID negative	N/A, negative	ND	**Maternal Plasma**: IgG HA; **Cord Plasma**: IgG HA	
68	COVID negative	N/A, negative	ND	**Maternal Plasma**: IgG HA; **Cord Plasma**: IgG HA	INFLAM
69	COVID negative	N/A, negative			INFLAM
70	COVID negative	N/A, negative			HYPO, OTHER
71	COVID negative	N/A, negative			GROSS, OTHER
72	COVID negative	N/A, negative			MVM
73	COVID negative	N/A, negative			GROSS
74	COVID positive	asymptomatic/mild	ND	**Maternal Plasma**: IgG RBD, IgG HA; **Cord Plasma**: IgG RBD, IgG HA	FVM, INFLAM, GROSS
75	COVID negative	N/A, negative			HYPO

© 2020 Edlow AG et al. *JAMA Network Open.*

Viral Load Analysis	Antibody Analysis	Placental Pathology	Viral Load Analysis	Antibody Analysis	Placental Pathology
76	COVID negative	N/A, negative			INFLAM, HYPO, GROSS
77	COVID positive	asymptomatic/mild	ND	**Maternal Plasma:** ND; **Cord Plasma**: IgG HA	GROSS
78	COVID positive	asymptomatic/mild	ND	**Maternal Plasma**: IgG HA; **Cord Plasma:** IgG HA	INFEC
79	COVID positive	asymptomatic/mild	ND	**Maternal Plasma**: ND; **Cord Plasma**: ND	FVM, INFLAM
80	COVID negative	N/A, negative			HYPO
81	COVID negative	N/A, negative			GROSS
82	COVID positive	asymptomatic/mild	ND		FVM, HYPO, GROSS, OTHER
83	COVID positive	moderate	ND	**Maternal Plasma**: ND; **Cord Plasma**: ND	FVM, INFLAM, OTHER
84	COVID negative	N/A, negative			FVM
85	COVID negative	N/A, negative			FVM, INFEC
86	COVID positive	severe	**Nasal Swab:** ND; **Oral Swab:** ND; **Sputum:** 3.4; **Maternal Plasma:** ND		
87	COVID negative	N/A, negative			NONE
88	COVID positive	asymptomatic/mild	ND	**Maternal Plasma**: ND; **Cord Plasma**: RBD IgG	INFLAM
89	COVID negative	N/A, negative			GROSS, OTHER
90	COVID negative	N/A, negative			MVM, OTHER
91	COVID negative	N/A, negative			INFEC, GROSS, OTHER

© 2020 Edlow AG et al. *JAMA Network Open.*

Participant number	COVID Status	COVID Severity	Viral Load Analysis	Antibody Analysis	Placental Pathology
92	COVID negative	N/A, negative			GROSS, OTHER
93	COVID negative	N/A, negative			HYPO, GROSS, OTHER
94	COVID negative	N/A, negative			FVM, HYPO, GROSS
95	COVID positive	asymptomatic/mild	ND	**Maternal Plasma**: IgG HA; **Cord Plasma**: IgG HA	
96	COVID positive	severe	ND	**Maternal Plasma:** IgG RBD, IgG N, IgG HA, IgM HA; **Cord Plasma**: IgG RBD, IgG HA	NONE
97	COVID positive	moderate	ND	**Maternal Plasma**: IgG RBD, IgG N, IgM RBD, IgG HA, IgM HA; **Cord Plasma**: IgG N, IgG HA	MVM
98	COVID positive	moderate	ND	**Maternal Plasma**: IgG RBD, IgG N, IgM N, IgG HA; **Cord Plasma**: IgG N, IgG HA	MVM, FVM
99	COVID positive	asymptomatic/mild	ND	**Maternal Plasma**: IgG HA, IgM HA; **Cord Plasma**: IgG HA	INFEC, OTHER
100	COVID negative	N/A, negative	ND	**Maternal Plasma**: IgG HA; **Cord Plasma**: IgG HA	
101	COVID positive	asymptomatic/mild	ND	**Maternal Plasma**: IgG HA; **Cord Plasma**: IgG HA	
102	COVID positive	asymptomatic/mild	ND	**Maternal Plasma**: IgG HA, IgM HA; **Cord Plasma**: IgG HA	OTHER
103	COVID negative	N/A, negative	ND	**Maternal Plasma**: IgG HA; **Cord Plasma**: IgG HA	
104	COVID positive	asymptomatic/mild	ND	**Maternal Plasma**: IgG RBD, IgG N, IgM N, IgG HA; **Cord Plasma**: IgG HA	INFEC
105	COVID negative	N/A, negative	ND	**Maternal Plasma**: IgG HA; **Cord Plasma**: IgG HA	
106	COVID negative	N/A, negative	ND	**Maternal Plasma:** IgG HA, IgM HA; **Cord Plasma:** IgG HA	

© 2020 Edlow AG et al. *JAMA Network Open.*

Participant number	COVID Status	COVID Severity	Viral Load Analysis	Antibody Analysis	Placental Pathology
107	COVID positive	asymptomatic/mild	ND	**Maternal Plasma**: IgG N, IgG HA, IgM HA; **Cord Plasma**: IgG RBD, IgG N, IgG HA	HYPO
108	COVID positive	asymptomatic/mild	ND		
109	COVID positive	severe	**Oral Swab:** 3.7; **Sputum:** 3.0; **Mat Plasma:** ND		
110	COVID positive	moderate	ND	**Maternal Plasma**: IgG RBD, IgG N, IgM RBD, IgM N, IgG HA, IgM HA; **Cord Plasma**: IgG RBD, IgG N, IgG HA	INFEC
111	COVID positive	asymptomatic/mild	ND		
112	COVID positive	severe	**Nasal Swab:** ND; **Oral Swab:** ND; **Sputum:** 2.4 (5/6/20), 2.8 (5/15); **Mat Plasma:** ND; **Placenta:** ND	**Maternal Plasma**: IgG RBD, IgG N, IgM N, IgG HA; **Cord Plasma**: IgG RBD, IgG N, IgG HA	MVM, HYPO, GROSS, OTHER
113	COVID positive	asymptomatic/mild	ND		
114	COVID positive	asymptomatic/mild	ND		
115	COVID positive	asymptomatic/mild	**Sputum:** 1.9		
116	COVID positive	asymptomatic/mild	ND		
117	COVID positive	asymptomatic/mild	ND		
118	COVID positive	asymptomatic/mild	ND		
119	COVID positive	asymptomatic/mild	ND		

© 2020 Edlow AG et al. *JAMA Network Open.*

Participant number	COVID Status	COVID Severity	Viral Load Analysis	Antibody Analysis	Placental Pathology
120	COVID positive	asymptomatic/mild	ND	**Maternal Plasma**: IgG RBD, IgG N, IgG HA; **Cord Plasma**: IgG RBD, IgG N, IgG HA	FVM, GROSS
121	COVID positive	asymptomatic/mild	ND		
122	COVID positive	asymptomatic/mild	ND		
123	COVID positive	asymptomatic/mild	ND	**Maternal Plasma**: IgG RBD, IgG N, IgM N, IgM HA; **Cord Plasma**: IgG RBD, IgG N, IgG HA	
124	COVID positive	asymptomatic/mild	ND	**Maternal Plasma**: ND; **Cord Plasma**: ND	INFEC, HYPO
125	COVID positive	asymptomatic/mild	ND		
126	COVID positive	asymptomatic/mild	ND	**Maternal Plasma:** IgG HA, IgM HA; **Cord Plasma:** IgG HA	INFLAM, OTHER
127	COVID positive	asymptomatic/mild	ND	**Maternal Plasma**: IgG N, IgG HA; **Cord Plasma**: IgG N, IgG HA	MVM, GROSS

[a]COVID-19 severity determined per modified National Institutes of Health (NIH) and Society for Maternal-Fetal Medicine criteria. *CB:* cord blood; *ND:* not detected. A blank space indicates the assay was not performed. For antibodies, antibodies above the background-corrected threshold of detection are listed. [b]Placental pathology abbreviations are listed in eTable 1.

© 2020 Edlow AG et al. *JAMA Network Open.*

eTable 4. Severe Maternal Morbidity

SARS-CoV-2 status	Maternal Morbidity	Age	Maternal Comorbidities	Pre-Pregnancy BMI	Maternal COVID Symptoms	Gestational age at delivery	Hypertensive disorder of pregnancy / Chronic HTN
positive, symptomatic	ARDS; ICU Admission, intubation	34	Thyroid disease, BMI > 30	34	Cough, fever/chills, shortness of breath, myalgia, fatigue, nausea, vomiting, headache	34.3	Yes
positive, asymptomatic	Hemorrhage, DIC	19	None	24.40	None	35.1	Yes
positive, symptomatic	Hemorrhage	38	None	26.84	cough, fever/chills, nausea/vomiting, other	38.4	No
positive, symptomatic	ARDS, ICU Admission, intubation	27	BMI>30; asthma	34.74	cough, fever/chills, shortness of breath, congestion, loss of taste, myalgia, fatigue, headache	still pregnant	
positive, symptomatic	Hemorrhage	28	None	23.05	other	40.6	No
positive, symptomatic	Hemorrhage	25	BMI>30	33.90	congestion, other	22.6	No
positive, asymptomatic	Hemorrhage	37	Thyroid disease, BMI > 30	30.90	none	40.2	No
positive, symptomatic	Hypoxia, lower respiratory tract disease, ICU Admission	35	None	25.03	cough, fever/chills, congestion, myalgia, other	37.9	No
positive, asymptomatic	Hysterectomy (placenta accreta)	33	Breast cancer	19.50	none	35.3	Yes
negative	Hemorrhage	33	None	18.70	None	42	No
negative	Hemorrhage	33	None	27.99	None	39.1	Yes

© 2020 Edlow AG et al. *JAMA Network Open.*

eTable 5. Severe Neonatal Morbidity

Maternal SARS-CoV-2 status	Gestational age at delivery (weeks)	Infant Sex	Infant Morbidity	Infant COVID status	Maternal Morbidity
positive, symptomatic	34.3	Female	CPAP, NICU, TTN	Negative	YES
positive asymptomatic	35.1	Male	Intrauterine Fetal Death	Not tested	YES
positive asymptomatic	38.9	Female	Supplemental O2, NICU, Pneumonia	Negative	NO
positive asymptomatic	34	Female	NICU	Negative	NO
positive asymptomatic	35.3	Male	NICU, CPAP, TTN	Negative	NO
positive, symptomatic	37.1	Female	NICU	Negative	NO
positive, symptomatic	35.6	Female	NICU, Assisted ventilation	Negative	NO
positive, symptomatic	35.1	Male	NICU	Negative	YES
positive, symptomatic	22	Male	Neonatal death; RDS	Not tested	YES
positive, symptomatic	31.4	Male	NICU, RDS, CPAP	Negative	NO
positive, symptomatic	41	Female	NICU	Negative	NO
positive, symptomatic	37.3	Female	NICU	Negative	NO
positive asymptomatic	35.3	Female	RDS; CPAP; NICU	Negative	YES
positive, symptomatic	37.4	Male	NICU	Negative	NO
negative	37.7	Male	CPAP, NICU, TTN	Not tested	NO
negative	34.6	Female	NICU	Not tested	NO
negative	37.1	Male	CPAP, NICU, TTN	Not tested	NO
negative	39.4	Male	CPAP, NICU, TTN	Not tested	NO
negative	42	Male	CPAP, NICU, TTN	Not tested	NO
negative	41.4	Male	NICU	Not tested	NO

CPAP: Continuous positive pressure airway pressure; **NICU:** Neonatal intensive care unit admission; **RDS:** respiratory distress syndrome; **TTN:** Transient Tachypnea of the Newborn

© 2020 Edlow AG et al. *JAMA Network Open.*

eTable 6. Participant Characteristics, Viral Loads, Antibody Quantification, and Placental Pathology Stratified by Maternal Disease Severity

	SARS-CoV-2 positive asymptomatic/mild (n=45)	SARS-CoV-2 positive mod/severe/critical (n=19)	P-value
**Preterm delivery[a]**	6 (14%)	5 (71%)	0.21
**Trimester at diagnosis**			
Second	3 (7%)	6 (32%)	0.02
Third	42 (93%)	13 (68%)	
**Detectable viral load[b]**	5 (11%)	6 (32%)	0.04
**Highest viral load (mean, SD)**	2.91 (1.27)	4.51 (1.42)	0.08
**Maternal antibody results[c]**			
Time from symptom onset to study blood draw (mean, SD)	23.04 (20.10)	32.6 (26.5)	0.20
Maternal anti-IgG RBD (mean, SD)	0.33 (0.48)	0.55 (0.61)	0.23
Maternal anti-IgG N (mean, SD)	0.74 (0.72)	1.16 (1.09)	0.21
**Therapy**			
Remdesivir	--	3 (16%)	--
Hydroxychloroquine	--	3 (16%)	--
**Placental Pathology[d]**			
MVM	9 (30%)	7 (54%)	0.14

MVM: maternal vascular malperfusion-associated lesions.
[a]Among patients who delivered at the time of analysis (N=42 ASX/mild patients delivered; N=14 mod/severe/critical delivered); [b]Among N=62 who had viral load analysis performed (N=44 ASX/mild, N=18 mod/severe/critical); [c]Among N=39 SARS-CoV-2 positive who had antibody levels analyzed (N=27 ASX/Mild, N=12 mod/severe/critical); [d] Among N=44 patients who had placental pathology performed (N=30 Asymptomatic/Mild, N=14 mod/severe/critical). P-values for proportions calculated using chi-square or Fisher’s exact test, Mann-Whitney U used to calculate p-values for continuous variables.

© 2020 Edlow AG et al. *JAMA Network Open.*

eFigure 3. Maternal Viral Load by Time Elapsed From SARS-CoV-2 Diagnosis

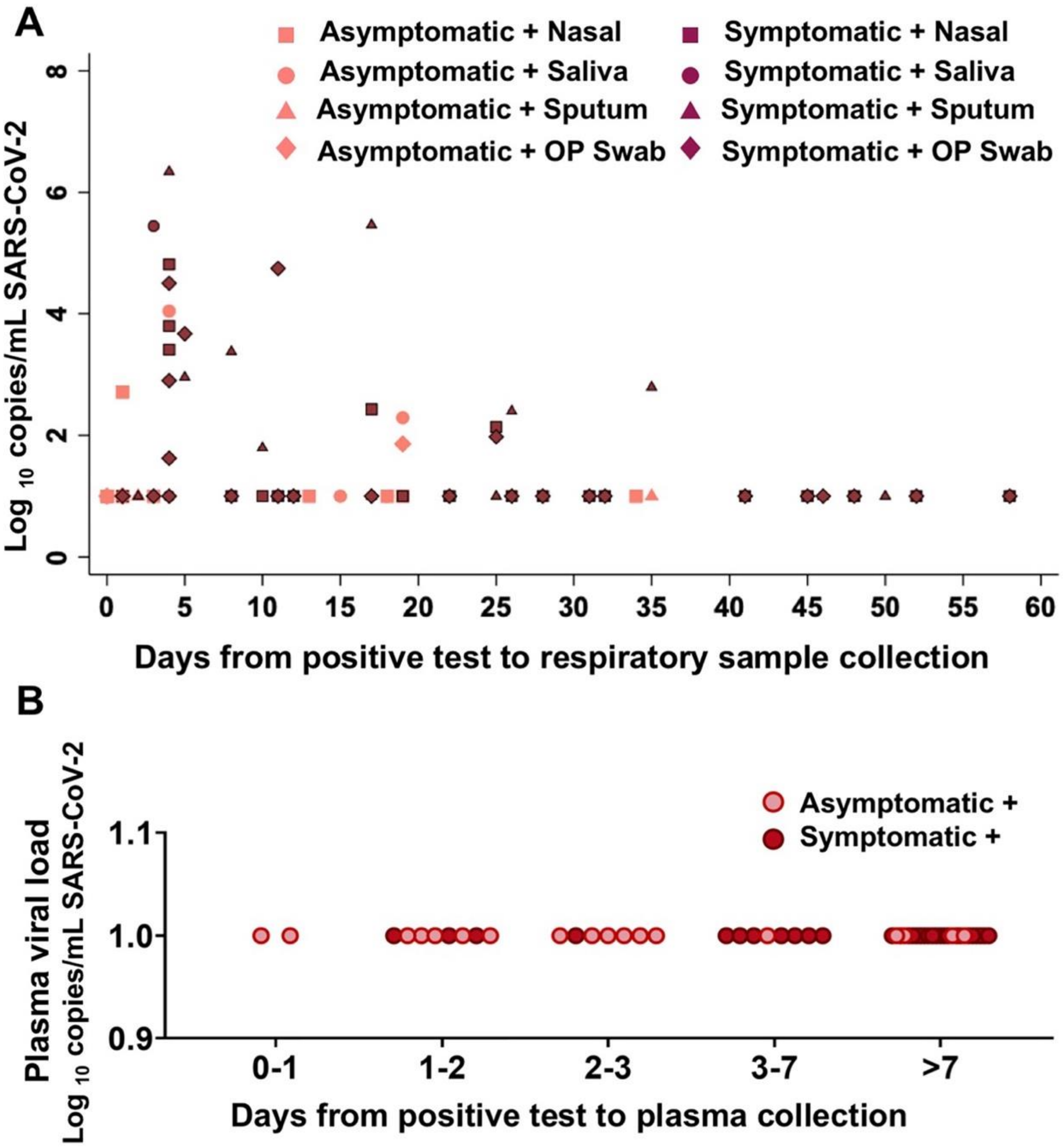

eFigure 3A/B: Viral load in maternal respiratory samples (A) and maternal plasma (B) by days elapsed from SARS-CoV-2 diagnosis to study sample collection.
Asymptomatic patients are depicted in light red, and symptomatic in dark red.
(A) 44 SARS-CoV-2 positive patients provided samples for respiratory viral load quantification. The median time from SARS-CoV-2 diagnosis to collection of first respiratory specimen was 9 days (IQR: 1-26.5 days).
(B) 53 SARS-CoV-2 positive patients provided samples for plasma viral load quantification**.** Median time from SARS-CoV-2 diagnosis to first blood draw for viral load patients was 9 days (IQR 2-25 days). Elapsed time between diagnosis and donation of study samples occurred due to the natural course of the disease (often worsening symptoms requiring hospitalization 7-10 days after symptom onset), and also reflected patient preferences regarding which samples to give and when during their hospitalization.

© 2020 Edlow AG et al. *JAMA Network Open.*

eTable 7. Detectable Viral Load and Maternal and Neonatal Antibody

	No detectable viral load (N=51)	Detectable viral load (N=11)	P-value
**Antibody Analysis**			
Maternal IgG RBD	0.29 (0.36)	0.79 (0.91)	0.02
Neonatal IgG RBD	0.24 (0.31)	0.28 (0.50)	0.77
IgG RBD transfer ratio, mean (SD)	0.77 (0.60)	0.31 (0.29)	0.15
Maternal IgG N	0.84 (0.79)	0.92 (1.2)	0.85
Neonatal IgG N	0.69 (0.44)	0.78 (1.1)	0.96
IgG N transfer ratio, mean (SD)	0.70 (0.44)	0.75 (0.31)	0.84

P-values generated from Mann Whitney U test

© 2020 Edlow AG et al. *JAMA Network Open.*

eTable 8. Correlations Between Maternal and Neonatal Antibody Titers, Transplacental Antibody Transfer, and Maximum Maternal Viral Load

	**Spearman rho correlation coefficient with maximum maternal viral load**	**P-value**
Background-corrected neonate IgG RBD	-0.96	0.0005
Background-corrected neonate IgG N	-0.94	0.005
Background-corrected maternal IgG RBD	-0.70	0.08
Background-corrected maternal IgG N	-0.88	0.02
IgG RBD transfer ratio	-1.00	<0.001
IgG N transfer ratio	-1.00	<0.001

Transfer ratio defined as cord antibody titer (OD450-570)/maternal antibody titer. As maximum maternal viral load increases, maternal IgG titers against SARS-CoV-2 N-antigen are reduced, as are umbilical cord titers of anti-RBD IgG and N IgG. Transplacental antibody transfer for RBD and N is also significantly reduced as maximum maternal viral load increases. P-values generated by Spearman rank correlation.

© 2020 Edlow AG et al. *JAMA Network Open.*

eTable 9. Antibody Transfer Ratio in Full-Term vs Preterm Gestations

	IgG RBD transfer ratio[b] (mean ±SD)	IgG N transfer ratio[c] (mean ±SD)	IgG HA transfer ratio[d] (mean ±SD)
Full term[a]	0.82 ± 0.57	0.85 ± 0.47	1.72 ± 1.44
Preterm	0.27 ± 0.33	0.25 ± 0.59	1.33 ± 0.72

[a]Full term is ≥ 37 weeks, preterm is < 37 weeks.[b]N=20 for full term and 4 for preterm (SARS-CoV-2 positive only). [c]N= 22 for full term and 4 for preterm (SARS-CoV-2 positive only). [d] 75/77 mothers had detectable anti-HA titers, 63 full term and 12 for preterm. There was a trend toward reduced transfer of anti-HA antibody in preterm neonates, p=0.08 by Mann-Whitney U. P values not calculated for RBD and N, given small Ns.

© 2020 Edlow AG et al. *JAMA Network Open.*

eFigure 4. Maternal Antibody Titers by Days From Symptom Onset

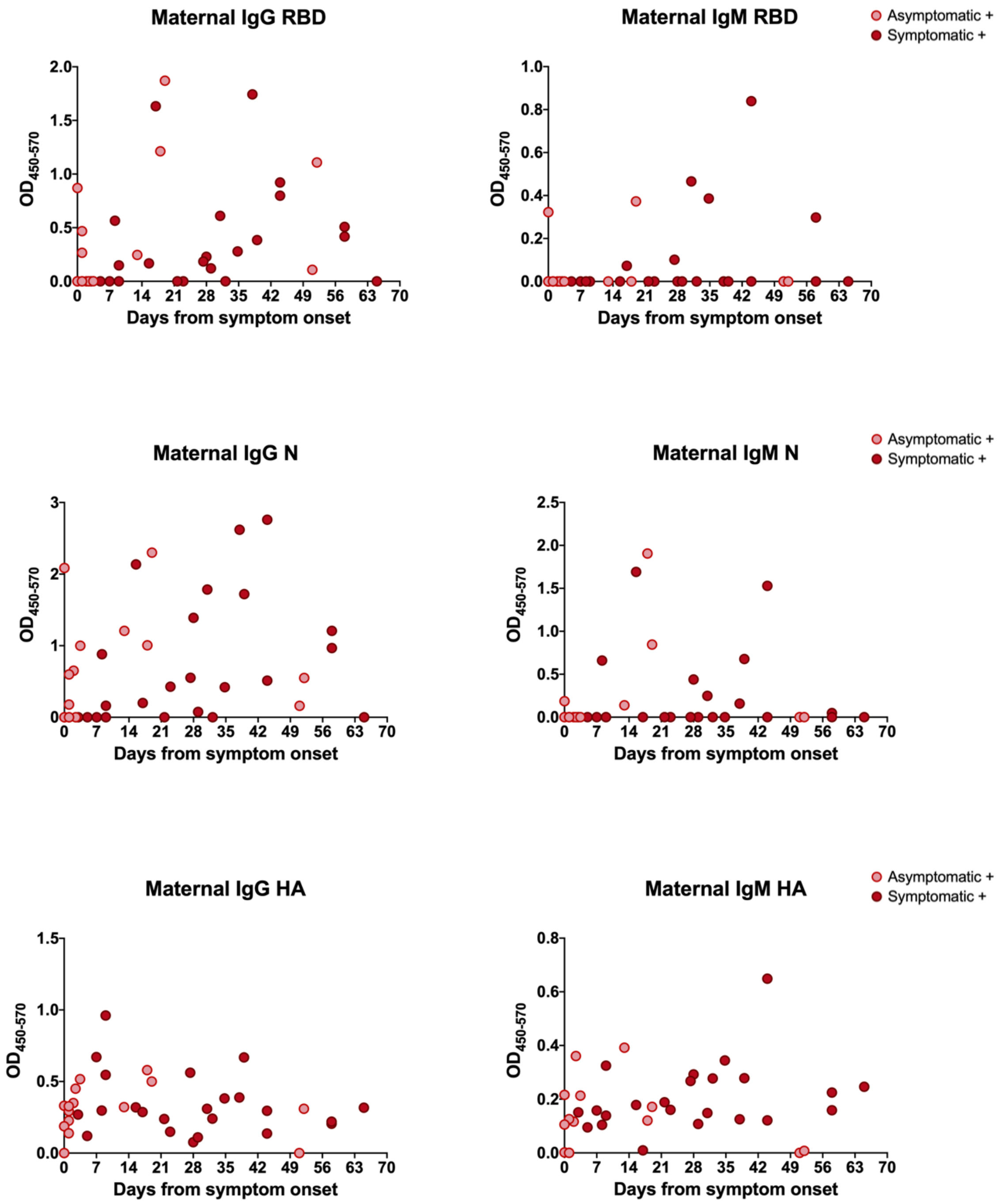

eFigure 5 and 6: Maternal (A) and cord blood (B) antibody titers by days from maternal symptom onset.
IgG against RBD, N and HA depicted on left, IgM on right. Titers are represented as the OD450 value subtracted from the reference OD570 value and are shown as the average of two replicates. For asymptomatic patients (light red circles), the interval from “symptom onset” to study blood draw was defined as interval from positive SARS-CoV-2 test to maternal blood draw (unable to determine duration of illness). The mean time from symptom onset to blood draw for antibody quantification among symptomatic individuals was 28.6 $\pm$ 18.1 days. Many asymptomatic participants have detectable antibody titer, suggesting duration of illness even greater than the estimated interval. Spearman correlations between duration of symptoms and antibody titers amongst symptomatic patients: Maternal IgG RBD: rho=0.42, p=0.03; Maternal IgM RBD: rho=0.21, p=0.31; Maternal IgG N: rho=0.43, p=0.05; Maternal IgM N: rho=0.17, p=0.46; Cord IgG RBD: rho=0.42, p=0.03; Cord IgG N: rho=0.50, p=0.02. Of the 12 SARS-CoV-2 positive women with no detectable anti-RBD or anti-N antibody, eight were symptomatic. 3/12 SARS-CoV-2 positive women with undetectable anti-RBD or anti-N antibodies were preterm (<37 weeks). Four of 12 had >14 days elapse from time of symptom onset to blood draw, the remaining 8 were either asymptomatic or had <7 days from symptom onset to blood draw.

© 2020 Edlow AG et al. *JAMA Network Open.*

eFigure 5. Cord Antibody Titers by Days from Maternal Symptom Onset

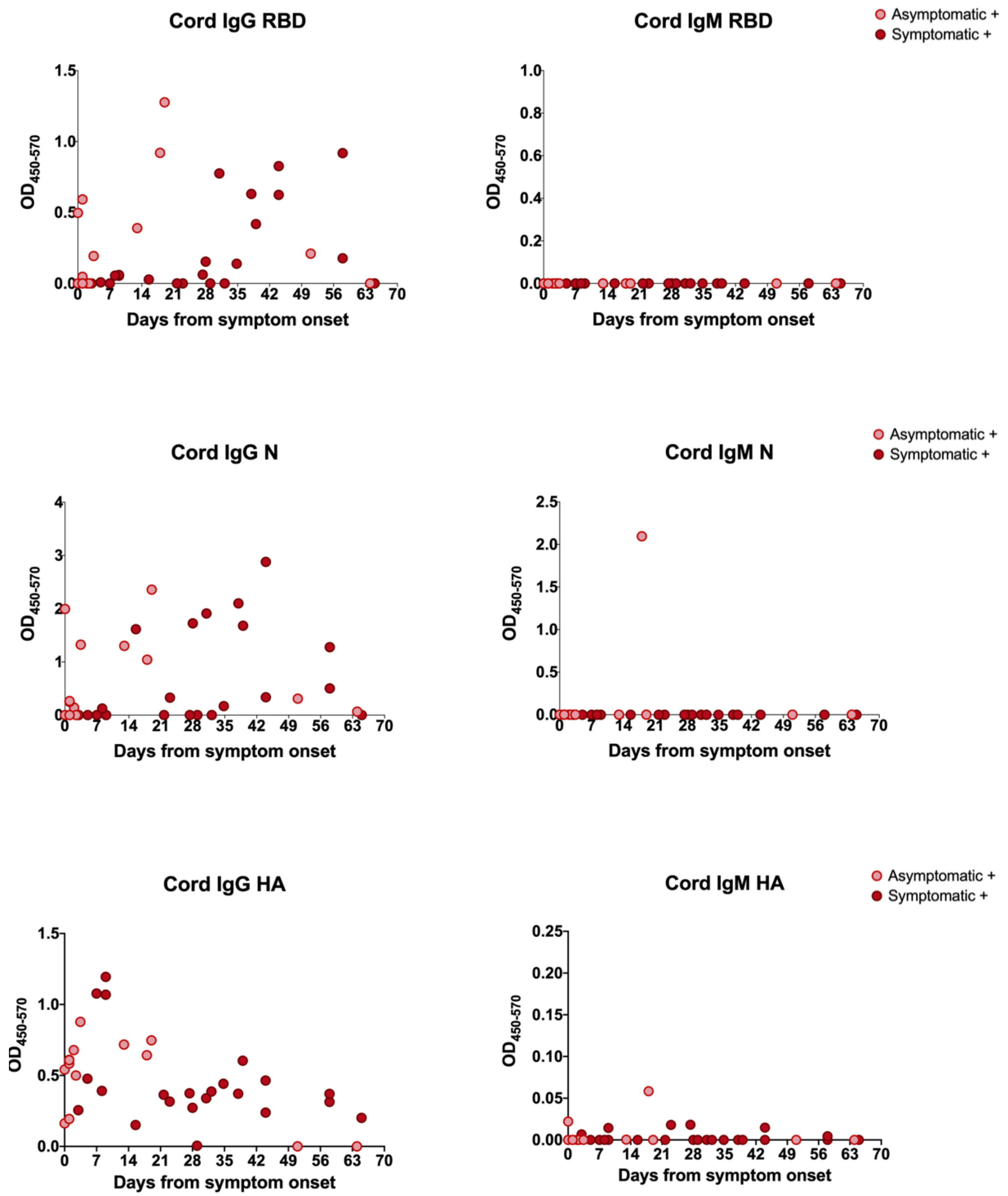

eFigure 5 and 6: Maternal (A) and cord blood (B) antibody titers by days from maternal symptom onset. IgG against RBD, N and HA depicted on left, IgM on right. Titers are represented as the OD450 value subtracted from the reference OD570 value and are shown as the average of two replicates. For asymptomatic patients (light red circles), the interval from “symptom onset” to study blood draw was defined as interval from positive SARS-CoV-2 test to maternal blood draw (unable to determine duration of illness). The mean time from symptom onset to blood draw for antibody quantification among symptomatic individuals was 28.6 $\pm$ 18.1 days. Many asymptomatic participants have detectable antibody titer, suggesting duration of illness even greater than the estimated interval. Spearman correlations between duration of symptoms and antibody titers amongst symptomatic patients: Maternal IgG RBD: rho=0.42, p=0.03; Maternal IgM RBD: rho=0.21, p=0.31; Maternal IgG N: rho=0.43, p=0.05; Maternal IgM N: rho=0.17, p=0.46; Cord IgG RBD: rho=0.42, p=0.03; Cord IgG N: rho=0.50, p=0.02. Of the 12 SARS-CoV-2 positive women with no detectable anti-RBD or anti-N antibody, eight were symptomatic. 3/12 SARS-CoV-2 positive women with undetectable anti-RBD or anti-N antibodies were preterm (<37 weeks). Four of 12 had >14 days elapse from time of symptom onset to blood draw, the remaining 8 were either asymptomatic or had <7 days from symptom onset to blood draw.

© 2020 Edlow AG et al. *JAMA Network Open.*

eTable 10. Placental Pathology by Maternal SARS-CoV-2 Status

Placental Pathologic Category	SARS-CoV-2 negative controls (n=44) N (%)	SARS-CoV-2 positive cases (n=44) N (%)	P-values
Maternal Vascular Malperfusion	8 (18 %)	16 (36%)	0.06
Fetal Vascular Malperfusion	9 (20%)	10 (23%)	0.80
Infectious	5 (11%)	12 (27%)	0.06
Inflammatory	10 (23%)	14 (32%)	0.34
Hypoperfusion-related lesions	14 (32%)	11 (25%)	0.48
Gross	19 (43%)	9 (21%)	0.03
Other	14 (32%)	18 (41%)	0.38
Placental weight <=10th percentile	20 (45%)	26 (59%)	0.20

P-values generated from Pearson's chi square test. The composite outcome of any maternal vascular malperfusion, fetal vascular malperfusion, inflammatory (chronic villitis/villitis of unknown etiology) or infectious (histologic chorioamnionitis) finding was significantly increased in participants positive for SARS-CoV-2 (N=37/44 or 84%, versus 24/44 or 55% in SARS-CoV-2 unexposed), chi-squared p= 0.003. Examining days elapsed from maternal symptom onset to delivery as a continuous variable, there was no difference in rate of placental pathology.

© 2020 Edlow AG et al. *JAMA Network Open.*

eTable 11. Placental Pathology by Maternal SARS-CoV-2 Severity

**Placental Pathologic Category**	**SARS-CoV-2 negative controls (n=44) N (%)**	**SARS-CoV-2 positive Asymptomatic/ mild (n=30) N (%)**	**SARS-CoV-2 positive moderate/severe / critical cases (n=14) N (%)**	**P-value**
Maternal Vascular Malperfusion	8 (18%)	9 (30%)	7 (50%)	0.06[a]
Fetal Vascular Malperfusion	9 (20%)	8 (27%)	2 (14%)	0.63
Infectious	5 (11%)	9 (30%)	3 (21%)	0.13
Inflammatory	10 (23%)	12 (40%)	2 (14%)	0.13
Hypoperfusion-related lesions	14 (32%)	7 (23%)	4 (29%)	0.73
Gross	19 (43%)	7 (23%)	2 (15%)	0.07
Other	14 (32%)	11 (37%)	7 (50%)	0.47
Placental weight <=10th percentile	20 (45%)	17 (57%)	9 (64%)	0.39

[a] A SARS-CoV-2 positive mother was more likely to have maternal vascular malperfusion lesions as disease severity increased (OR 2.09 (95% CI 1.11-3.97) using a univariate logistic regression model to test the hypothesis of an ordered relationship between increasing SARS-CoV-2 severity and maternal vascular malperfusion on placental pathology, p=0.02). Remainder of p-values generated from Pearson's chi square test, with Bonferroni correction (adjusted p-value threshold p<0.025).

© 2020 Edlow AG et al. *JAMA Network Open.*

eReferences

1. Harris PA, Taylor R, Thielke R, Payne J, Gonzalez N, Conde JG. Research electronic data capture (REDCap)--a metadata-driven methodology and workflow process for providing translational research informatics support. J Biomed Inform 2009;42:377-81.
2. Shook LL, Shui JE, Boatin AA, et al. Rapid establishment of a COVID-19 perinatal biorepository: early lessons from the first 100 women enrolled. BMC Med Res Methodol 2020;20:215.
3. Research Use Only 2019-Novel Coronavirus (2019-nCoV) Real-time RT-PCR Primers and Probes. Centers for Disease Control and Prevention (CDC); Accessed April, 2020.
4. Nguewa PA, Agorreta J, Blanco D, et al. Identification of importin 8 (IPO8) as the most accurate reference gene for the clinicopathological analysis of lung specimens. BMC Mol Biol 2008;9:103.
5. Pizzolla A, Wang Z, Groom JR, et al. Nasal-associated lymphoid tissues (NALTs) support the recall but not priming of influenza virus-specific cytotoxic T cells. Proc Natl Acad Sci U S A 2017;114:5225-30.
6. Riemer AB, Keskin DB, Reinherz EL. Identification and validation of reference genes for expression studies in human keratinocyte cell lines treated with and without interferon-gamma - a method for qRT-PCR reference gene determination. Exp Dermatol 2012;21:625-9.
7. Palmer S, Wiegand AP, Maldarelli F, et al. New real-time reverse transcriptase-initiated PCR assay with single-copy sensitivity for human immunodeficiency virus type 1 RNA in plasma. J Clin Microbiol 2003;41:4531-6.
8. Khong TY, Mooney EE, Ariel I, et al. Sampling and Definitions of Placental Lesions: Amsterdam Placental Workshop Group Consensus Statement. Arch Pathol Lab Med 2016;140:698-713.
9. Saatcioglu HD, Kano M, Horn H, et al. Single-cell sequencing of neonatal uterus reveals an Misr2+ endometrial progenitor indispensable for fertility. Elife 2019;8.
10. Wang F, Flanagan J, Su N, et al. RNAscope: a novel in situ RNA analysis platform for formalin-fixed, paraffin-embedded tissues. J Mol Diagn 2012;14:22-9.
11. Chauhan SP, Rice MM, Grobman WA, et al. Neonatal Morbidity of Small- and Large-for-Gestational-Age Neonates Born at Term in Uncomplicated Pregnancies. Obstet Gynecol 2017;130:511-9.
12. Manuck TA, Rice MM, Bailit JL, et al. Preterm neonatal morbidity and mortality by gestational age: a contemporary cohort. Am J Obstet Gynecol 2016;215:103 e1- e14.
13. NIH COVID-19 Treatment Guidelines. 2020.
14. Management Considerations for Pregnant Patients With COVID-19 (updated 7/2/2020). 2020. (Accessed July 3, 2020, 2020, at https://s3.amazonaws.com/cdn.smfm.org/media/2415/SMFM_COVID_Management_of_COVID_pos_preg_patients_7-2-20.PDF_.pdf.)
15. COVID-19 Virus Infection and Pregnancy May 5, 2020. (Accessed September 21, 2020, 2020,
16. Coronavirus (COVID-19) Infection in Pregnancy: Information for Health Care Professionals. July 24, 2020. (Accessed September 25, 2020, 2020,

© 2020 Edlow AG et al. *JAMA Network Open.*
